# Fluorinated carbohydrates for ^18^F-positron emission tomography (PET)

**DOI:** 10.1039/d3cs00037k

**Published:** 2023-05-12

**Authors:** Emma Campbell, Christina Jordan, Ryan Gilmour

**Affiliations:** a Organisch-Chemisches Institut, Westfälische Wilhelms-Universität Münster Corrensstraße 36 48149 Münster Germany ryan.gilmour@uni-muenster.de; b Cells in Motion Interfaculty Centre, Westfälische Wilhelms-Universität Münster, Röntgenstraße 16 48149 Münster Germany

## Abstract

Carbohydrate diversity is foundational in the molecular literacy that regulates cellular function and communication. Consequently, delineating and leveraging this structure–function interplay continues to be a core research objective in the development of candidates for biomedical diagnostics. A totemic example is the ubiquity of 2-deoxy-2-[^18^F]-fluoro-d-glucose (2-[^18^F]-FDG) as a radiotracer for positron emission tomography (PET), in which metabolic trapping is harnessed. Building on this clinical success, more complex sugars with unique selectivities are gaining momentum in molecular recognition and personalised medicine: this reflects the opportunities that carbohydrate-specific targeting affords in a broader sense. In this Tutorial Review, key milestones in the development of 2-[^18^F]-FDG and related glycan-based radiotracers for PET are described, with their diagnostic functions, to assist in navigating this rapidly expanding field of interdisciplinary research.

## Abstract 2

In this Tutorial Review, key milestones in the development of 2-[^18^F]-FDG and related glycan-based radiotracers for PET are described.

## Graphical abstract

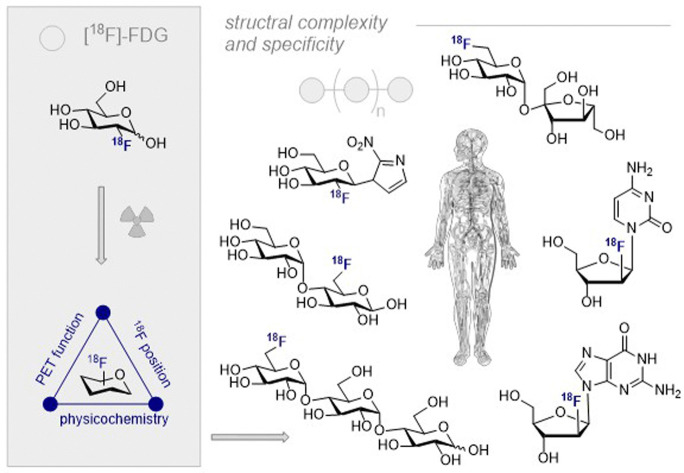

**Key learning points**

(1) 2-[^18^F]-FDG has been the gold standard PET tracer since the 1980s, but its lack of specificity limits clinical application.

(2) The structure–activity profile of 2-[^18^F]-FDG is instructive to design blueprints for future carbohydrate tracer development.

(3) Judicious selection of the carbohydrate core can enable increased specificity over 2-[^18^F]-FDG for individual disease cases.

(4) The site and configuration of the ^18^F-radiolabel provides a handle to modulate biological uptake and function.

## 1. Introduction

Advancing the field of personalised medicine is contingent on the development of effective diagnostic platforms to guide precision therapies.^1^ In the current suite of non-invasive imaging methods, positron emission tomography (PET) has emerged as a key enabling technology to investigate anatomy and metabolic function:^2^ this provides a powerful impetus to expand the existing repertoire of tailored PET tracers to meet the clinical demand. Of the existing small molecule portfolio, the ^18^F-fluorinated monosaccharide, 2-deoxy-2-[^18^F]-fluoro-d-glucose (1) (2-[^18^F]-FDG or [^18^F]FDG),^3–6^ is most broadly utilised, and largely responsible for the transformative success of PET worldwide. Grounded in the translational efficacy of fluorine bioisosterism *in vivo*,^7–11^ this exemplar of molecular mimicry enables cellular glycolysis to be expropriated thereby ensuring that 2-[^18^F]-FDG (1) reaches metabolically upregulated cells. In contrast to native d-glucose (2), OH → ^18^F substitution at C2 prohibits further cell metabolism, thereby ensuring accumulation of the radiotracer.^12^ Upon decay, positron emission occurs relatively quickly (*t*_1/2_  =  109.8 minutes to generate ^18^O) and in a highly localised environment. The annihilation event resulting from collision of a positron and an electron produces γ-rays, which are emitted in a 180° relationship: these γ-rays can be reconstituted thereby providing clinicians with detailed topographical information about the affected tissue. Buoyed by the clinical impact of 2-[^18^F]-FDG (1), a broad spectrum of specialised carbohydrate-based radiotracers has been established for applications in nuclear medicine.^13^ In this Tutorial Review, a systematic, structure-based overview of ^18^F-modified sugars is disclosed together with relevant physico- and radio-chemical data. This classification strategy is intended to provide a practical structure–function guide to carbohydrate-based radiotracer development and stimulate further interest in this cornerstone of personalised medicine.

**Fig. 1:**
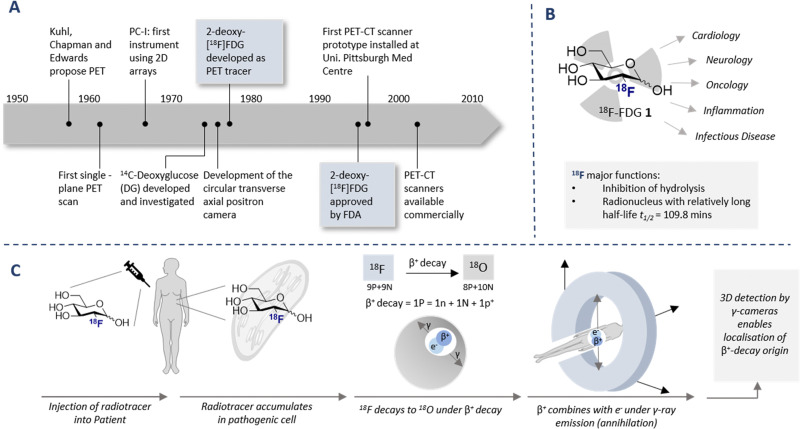
(A) Timeline of key historical milestones in the development of positron emission tomography (PET). (B) Schematic of 2-[^18^F]-FDG (1) and its use in various diagnostic fields. (C) Timeline of PET analysis: intravenous injection of a patient with ^18^F labelled radiotracer. The radiotracer accumulates in the target cell as it cannot be fully metabolised due to the presence of the C(sp^3^)–F (metabolic trapping), and subsequently decay to ^18^O (through β^+^ decay) is observed. Collision with a positron results in the emission of γ-rays in a 180° angle. The γ-rays are detected by a circular 3D γ-cameras allowing an image to be generated.

The diagnostic paradigm of PET has its origins in the early work Kuhl, Chapman and Edwards in the late 1950s (Fig. 1A).^14^ Tomography imaging by Ter-Pogossian and Phelps followed,^15,16^ and the rapid growth of the field, including the introduction of the single plane PET instrument, culminated in the validation of the circular transverse positron camera.^17,18^ These technological advances fuelled the search for suitable synthetic (radiolabeled) molecules that would facilitate full body imaging: a seminal advance was the validation of ^14^C-deoxyglucose in autoradiography.^19^ This culminated in the discovery of one of the most important diagnostic compounds of the last century, 2-[^18^F]-FDG (1) (Fig. 1B). Following the synthesis of the (cold) ^19^F counterpart 2-FDG,^20^ the synthesis of (hot) 2-[^18^F]-FDG (1) was first reported by Wolf and co-workers in 1978.^21^ Clinical translation of this tracer was contingent on establishing that 2-[^18^F]-FDG (1) was an acceptable substrate for hexokinase, and that metabolic trapping occurred (*i.e.* further glycolysis was paused). Since these conditions were met, tissue accumulation occurred thereby rendering 2-[^18^F]-FDG (1) an excellent candidate for d-glucose transport imaging.^22^ Since its conception and introduction to the clinic, 2-[^18^F]-FDG (1) has revolutionised *in vivo* PET imaging: this has been summarised (up until 2001) in an excellent review by Gambhir *et al.*^23^

**Scheme 1:**
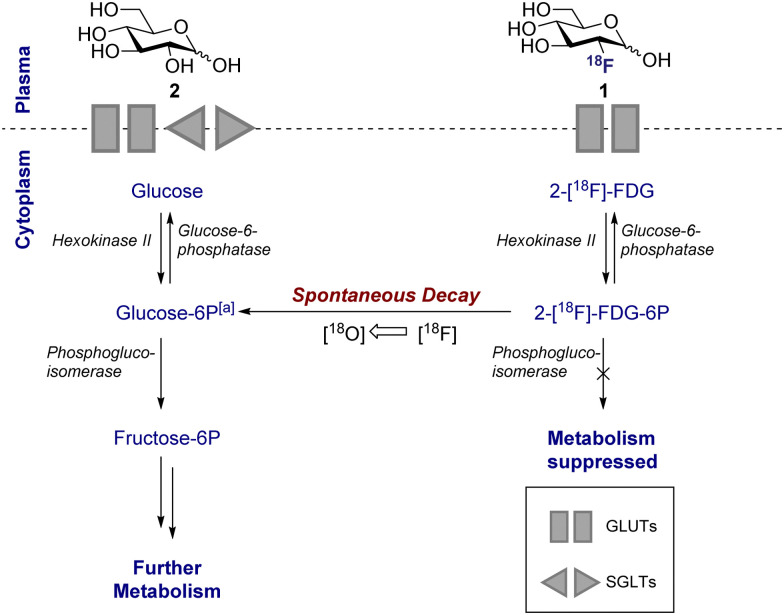
Schematic of d-glucose *versus* 2-[^18^F]-FDG metabolism. Key shows which transport proteins facilitate cellular uptake. [a] Decay of 2-[^18^F]-FDG produces d-glucose-6P with a heavy ^18^O in the 2-position.

The parallels between PET imaging and photography are striking, but in the former case the 3D-camera is reliant on gamma rays emitted from the point of interest within the body, rather than light. The generation of this gamma signature is conditional on injection of a radiotracer (in this case ^18^F) into the patient and subsequent efficient distribution (Fig. 1C). In the case of 2-[^18^F]-FDG (1), accumulation is a consequence of molecular editing with fluorine in which a subtle, bioisoteric OH to F replacement suppresses glycolysis. Subsequent β^+^ decay converts the radioisotopic ^18^F to ^18^O by emitting both a neutrino (n) and a positron (β^+^). The latter species is short lived and, upon collision with an electron, undergoes an annihilation event with the emission of two photons of equal energy (511 keV each) in a 180° angle. The recurring emission can be detected and reconstituted to construct a 3D image that conveys the origins of positron emission. Moreover, the ultimate decay of the ^18^F of 2-[^18^F]-FDG (1) to ^18^O ensures that, upon protonation, native d-glucose (2) is generated and the conventional glycolysis pathway continues (Scheme 1). Generating d-glucose (2) as the sole metabolite of 2-[^18^F]-FDG (1) injection, coupled with the low molar activities used in PET, mitigates concerns pertaining to side effects and enables diagnostic information to be gleaned by pausing a cellular process.

Application of 2-[^18^F]-FDG (1) as a diagnostic tool has proven to be expansive owing to the importance of d-glucose (2) as a cellular energy source and the metabolic disparity that differentiates healthy and abnormal cells. Pioneering work by Warburg and Minami in the early 1920s laid the foundations for the venerable “Warburg effect”: the phenomenon that high levels of anaerobic glycolysis is observed in tumour cells.^24,25^

**Fig. 2:**
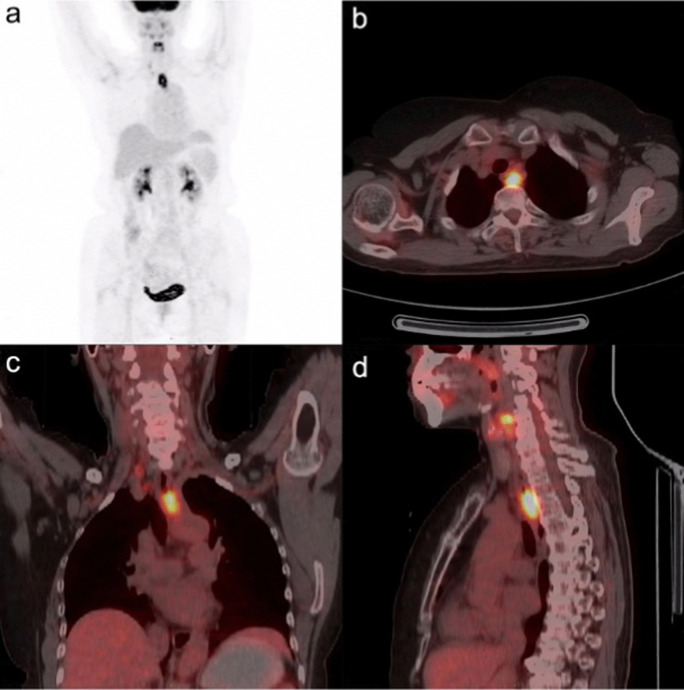
^18^F-FDG PET/CT fusion image of esophageal squamous cell carcinoma in a 68-years-old woman. The SUV of the tumour was determined to be 3.75 and the metabolic tumour volume was 0.155 m^−1^. This research was originally published in Radiation Oncology: L. Xia, L., X. Li., J. Zhu, Z. Gao, J. Zhang, G. Yang, Z. Wang, Prognostic value of baseline ^18^F-FDG PET/CT in patients with esophageal squamous cell carcinoma treated with definitive (chemo)radiotherapy. *Radiat. Onco.*, 2023, **18**, 41. Reused under the Creative Commons Attribution 4.0 International License (https://creativecommons.org/licenses/by/4.0/).

Warburg subsequently received the 1931 Nobel Prize in Physiology for Medicine “*for his discovery of the nature and mode of action of the respiratory enzyme*”. An example of the effectiveness of 2-[^18^F]-FDG PET/CT in visualising esophageal squamous cell carcinoma is shown in Fig. 2.

This disparity in metabolic phenotypes can be leveraged to distinguish tumour cells *in vivo* by PET imaging.^26,27^ In the conventional glycolysis pathways, the internalisation of d-glucose (2) into the cell is regulated by two d-glucose transporters (GLUT-1 and GLUT-3), which then enables subsequent phosphorylation by the enzyme hexokinase II (HK-II).^22,27^ The phosphorylated d-glucose (Glucose-6P) is then isomerised to fructose-6P, enabling further metabolism (Scheme 1). The molecular mimic, 2-[^18^F]-FDG (1), initially participates in the same uptake process, but subsequent glycolysis is paused by fluorine substitution at the C2-OH. This single atom edit is not compatible with phosphogluco-isomerase, leading to trapping and accumulation of ^18^F-labelled d-glucose within the cell. It is interesting to note that any remaining 2-[^18^F]-FDG (1) which is not phosphorylated to 2-[^18^F]-FDG-6-P by the hexokinase is excreted: this lowers the background signal which, in turn, improves the image resolution.^28^ Furthermore since glucose-6-phosphatase, which regulates dephosphorylation, is under-expressed in tumour cells, tracer accumulation is further amplified.^29^

The mechanism of accumulation is multifaceted and may be considered as a combination of d-glucose metabolism, hexokinase and d-glucose-6-phosphate activity, and the excretory pathway through the urinary tract. Despite this complexity, 2-[^18^F]-FDG (1) uptake is known to be proportional to d-glucose consumption.

Collectively, the introduction of the ^18^F-label at the 2-position of d-glucose serves two principal functions: (i) it mimics the natural abundance isotope,^30^ thereby inhibiting d-glucose metabolism and facilitating accumulation in affected cells, and (ii) it serves as an imaging probe to study organ dysfunction. Deconstructing and harnessing this unique, structure-specific mechanism (*vide infra*) enables an array of cancer pathologies to be imaged as well as providing a platform for investigating d-glucose metabolism, kinetics and inflammation.

## 2. Synthetic routes to 2-[^18^F]-FDG: a brief history

### 2.1 Electrophilic fluorination

**Scheme 2:**
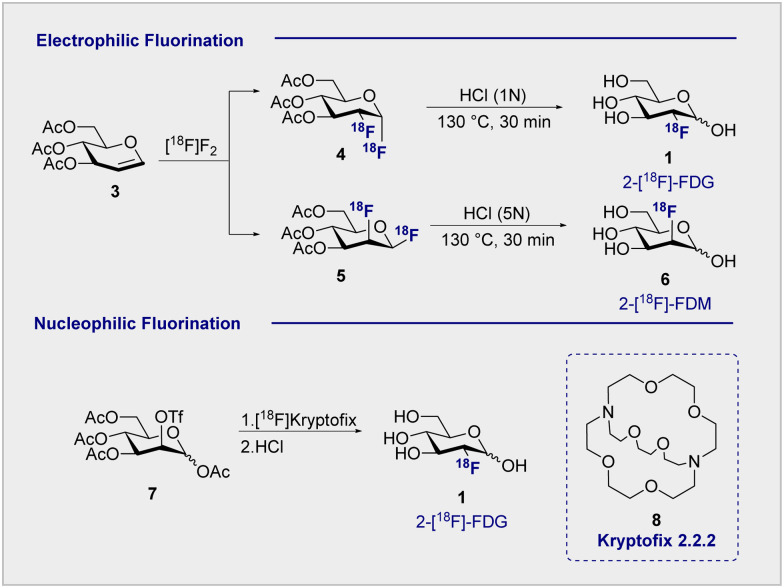
^18^F Fluorination methods: (upper) electrophilic fluorination produces both 2-[^18^F]-FDG (1) and 2-[^18^F]-FDM (6) products; (lower) nucleophilic fluorination produces solely glucose-configured 2-[^18^F]-FDG (1).

Functional small molecule validation is a powerful driver of synthetic innovation and the validation of 2-[^18^F]-FDG (1) in the context of PET is no exception. Early synthetic routes to these fluorinated monosaccharides relied on direct electrophilic fluorination of 3,4,6-tri-*O*-acetylglucal with hazardous elemental fluorine, followed by global hydrolysis using HCl.^4,31^ Although successful, this protocol furnished 2-[^18^F]-FDG (1) and 2-deoxy-2-[^18^F]fluoro-d-mannose (6) (2-[^18^F]-FDM) in a 3 : 1 ratio (Scheme 2, upper). Fuelled by increasing demand for the radiotracer, a re-evaluation of the initial synthesis routes was required to accommodate the short half-life of the ^18^F isotope (*t*_1/2_ = 109.8 min). Whilst the half-life does permit limited synthetic manipulations to be conducted, the separation of diastereoisomeric mixtures represented a major bottleneck in production that required attention. Although the exploration of reagents such as [^18^F]-acetyl-hypofluorite led to an improvement in yield and selectivity (95% yield of the glucose tracer),^32,33^ the radiochemical yield (RCY = 24 ± 3%) remained low.^34^

### 2.2 Nucleophilic fluorination

Electrophilic paradigms proved essential in generating 2-[^18^F]-FDG (1) for early studies in mice, dogs and humans, and remained indispensable for two decades after the first synthesis was reported.^3,5^

However, the introduction of Kryptofix® [2.2.2] (8) to generate 2-[^18^F]-FDG (1) by nucleophilic displacement of the d-mannose-derived triflate by Hamacher *et al.* proved transformative (Scheme 2, lower).^35^ Whilst conceptually related nucleophilic approaches had already been communicated, a series of synthetic limitations remained unresolved, particularly poor deprotection or ^18^F incorporation.^36,37^ The addition of Kryptofix® [2.2.2] (8) was found to enhance fluoride nucleophilicity and, by extension, ^18^F incorporation.^38^ Under optimised conditions, the fluorination of 1,3,4,6-tetra-*O*-acetyl-2-*O*-trifluoromethanesulfonyl-β-d-manno-pyranose (7), followed by global deprotection using HCl, enabled 2-[^18^F]-FDG (1) to be generated with an uncorrected yield of 44 ± 4% (Scheme 2).^35^ Acidic hydrolysis with HCl, classically requiring elevated temperatures and longer reaction times, can be achieved at room temperature under basic conditions. This further circumvents complications arising from nucleophilic displacement frequently observed in acidic conditions. It is pertinent to note that production efficiency remains a significant contributor to the clinical success of 2-[^18^F]-FDG (1), which received FDA approval in 1999.^39^ Following the transformative success of Kryptofix® [2.2.2] (8) in nucleophilic radiofluorination protocols, the next 15 years of process development focussed heavily on the evaluation of alternative ^18^F sources, improving deprotection efficiency and identifying impurities.^40,41^ It wasn’t until the early 2000s that the radiofluorination repertoire was augmented to include enzymatic fluorination.

### 2.3 Enzymatic fluorination

**Scheme 3:**
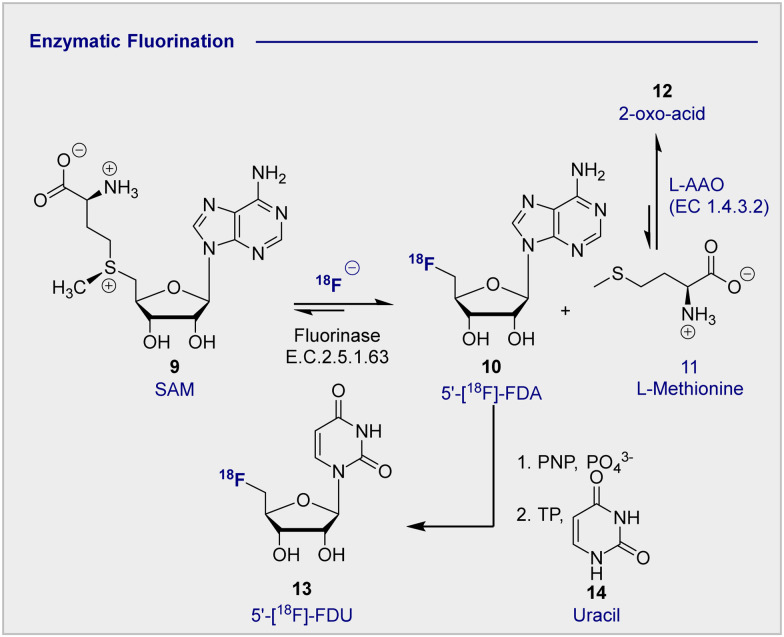
Enzymatic ^18^F fluorination enzyme to produce 5′-[^18^F]-FDA. 5′-[^18^F]-FDA can be further derivatised with PNP to produce 5′-[^18^F]-FDU.

In advancing the field of nucleophilic fluorination for PET, particularly in a clinical environment, post-reaction radiotracer isolation is a conspicuous consideration. The use of conventional organic solvents to enable fluorination must be reconciled with the need for aqueous solutions of the tracer to enable injection into the patient. Translating nucleophilic radiofluorination to an enzymatic paradigm would enable fluoride production, and C(sp^3^)–^18^F bond formation to be executed in the same aqueous phase, thereby significantly shortening operational times. In a seminal study, Prante and co-workers demonstrated the potential of this approach by coupling pre-labelled 2-[^18^F]-FDG (1) and UDP-2-[^18^F]-FDG using UDP-glucose-phosphorylase (EC 2.7.7.9, bovine liver).^42^ In the absence of a biocatalyst able to facilitate C–F bond formation, the application of enzymatic radiofluorination in PET seemed set to be restricted to transformations involving pre-fluorinated substrates. However, the isolation of 5′-fluorodeoxyadenosine synthase from *Streptomyces cattleya* by O’Hagan and co-workers proved to be a transformative event in organofluorine chemistry,^43,44^ and biocatalysis in a broader sense.^45^ The discovery of this first “fluorinase” not only allowed the biosyntheses of rare organofluorines to be rationalised,^46,47^ but it afforded unrivalled opportunities for the production of biomedically important materials.^47–49^ A totemic example of enzymatic radiotracer synthesis from the laboratories of Martarello and O’Hagan was the synthesis of radiolabelled 5′-deoxy-5′-[^18^F]-fluoroadenosine (10) (5′-[^18^F]-FDA) from commercially available *S*-adenosyl-methyltransferase (9) (SAM) in a RCY of up to 95% (Scheme 3).^50,51^ This remarkably high RCY was achieved through biasing the equilibrium of the reaction towards the product by judicious addition of l-amino acid oxidase (l-AAO). This addition of the second enzyme ensured that the by-product, l-methionine (11) was processed to the 2-oxo acid (12), thereby preventing the reverse reaction to regenerate the starting material. This enzymatic platform has been improved by over-expression of the gene encoding the catalyst in *Escherichia coli*. Enzymes are, in general, limited by their substrate specificity, however coupling with subsequent enzymes to affect downfield transformations (purine nucleoside phosphorylase (PNPs) and thymidine phosphorylases (TPs)), can broaden the scope immensely. Although biocatalysis-based fluorination strategies are highly appealing, leveraging this technology for clinical production remains in its infancy. A comprehensive survey of this area,^52^ and of methodological advances in synthetic radiochemistry in a more general sense,^53–56^ are beyond the scope of this review. Moreover, advances in modern automation approaches for the GMP clinical production of 2-[^18^F]-FDG (1) are discussed elsewhere.^57–62^

### 2.4 Exploring the stereochemical etiology of 2-deoxy-2-[^18^F]-fluoro-d-glucose (2-[^18^F]-FDG) effectiveness

**Fig. 3:**
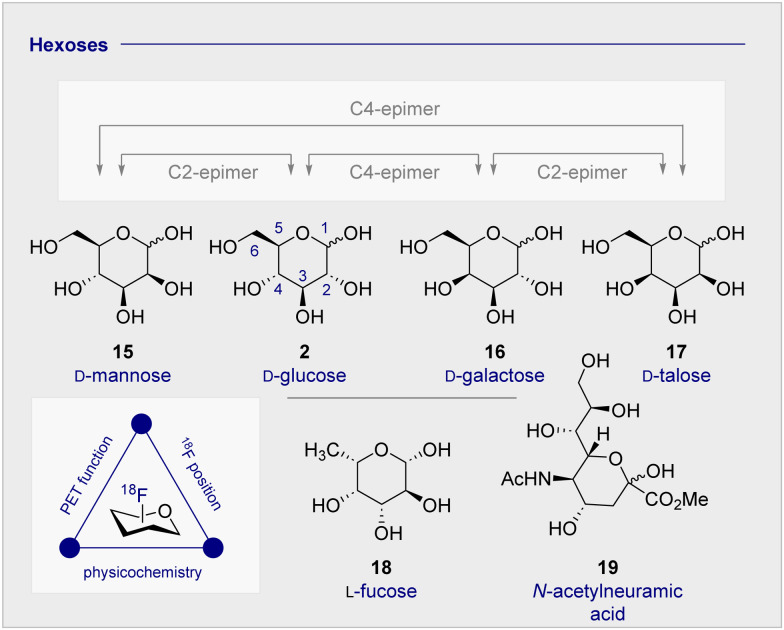
The relative configuration of selected monosaccharides.

The clinical validation of 2-[^18^F]-FDG (1), coupled with the relative stereochemical complexity of monosaccharides in general, continues to provide a strong foundation for the design and evaluation of structural analogues (*vide infra*). As it is illustrated in Fig. 3, many common hexoses are related by site-selective epimerisation at the C2 and/or C4 positions such that simple editing provides a handle to modulate the physicochemistry and function of the tracer. The effects of these seemingly subtle modifications often manifest themselves in human metabolism which, by extension, can be leveraged for diagnostic purposes: a pertinent example is the d-glucose (2), d-galactose (16), d-mannose (15) series.

Whilst d-mannose (15) and d-galactose (16) both occur frequently in nature and are important nutritional components, d-galactose (16) is particularly noteworthy as it can serve as a d-glucose (2) substitute, due to preferential consumption in the brain and associated insulin-independence.^63^ Indeed, studies in Wistar rats treated with streptozoticin have established that long-term oral d-galactose exposure reduces cognitive decline.^64^ This may have therapeutic implications for the management of neurodegenerative diseases, particularly in Alzheimer's disease where a high proportion of patients experience insulin-intolerance. Consequently, augmenting the small molecule tracer collection by structural editing, including regulatory deoxysugars (*e.g.*l-fucose (18))^65^ and neuraminic acid (19) derivatives^66,67^ continues to be intensively pursued.

## 3. ^18^F-modified monosaccharide tracers

### 3.1 An overview of the current portfolio of 2-[^18^F]-FDG-inspired monosaccharides for PET (Fig. 4)

**Fig. 4:**
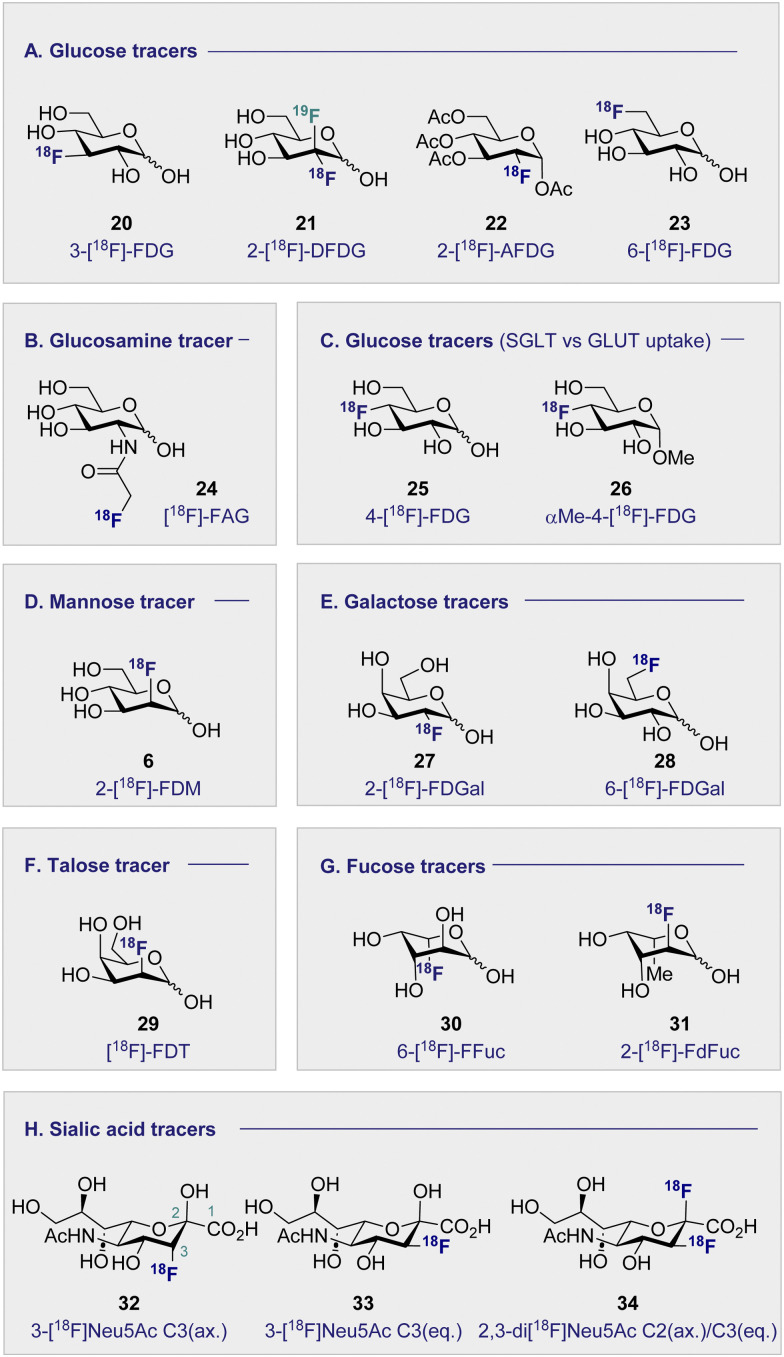
Structures of selected ^18^F monosaccharides.

Continued interest in 2-[^18^F]-FDG (1) stems not only from its clinical success, but also from a desire to understand the etiology of related hexoses in the context of PET. An early comparative analysis by Kearfott *et al.* in 1984 reported the effects of relocating the *quasi*-equatorial ^18^F-label by one position to generate 3-[^18^F]-fluoro-deoxy-d-glucose (20) (3-[^18^F]-FDG) (Fig. 4A).^68^

Investigations in mice, rats and dogs revealed that 2-[^18^F]-FDG (1) was consistently superior to 3-[^18^F]-FDG (20) in the heart, brain and kidneys.^68^ Furthermore, tissue retention was found to be higher with 2-[^18^F]-FDG (1), which is likely due to lower levels of phosphorylation and faster clearance of 3-[^18^F]-FDG (20) from the blood and kidneys. Regrettably, this unfavorable performance relative to 2-[^18^F]-FDG (1) has relegated 3-[^18^F]-FDG (20) from the list of preferred ^18^F-monosaccharides, and underscores the importance of label positioning in tracer design. Additional fluorination of the 2-[^18^F]-FDG (1) scaffold, to generate [^18^F]-2-deoxy-2,2-difluoro-d-glucose (21) (2-[^18^F]-DFDG) was destined to follow a similar fate.^69^ Whilst biodistribution in a Rhesus monkey was comparable to that observed with 2-[^18^F]-FDG (1), significant uptake in the surrounding muscles compromised the target to background ratio signal, thereby limiting further development of 2-[^18^F]-DFDG (21). From a preparative standpoint, leveraging globally protected monosaccharides for PET convey a range of advantages: the most notable include physicochemical modulation and the elimination of a time-consuming final deprotection step. In order to enhance the lipophilicity of 2-[^18^F]-FDG (1), and increase uptake rates, Waki *et al.* synthesised 1,3,4,6-tetra-acetyl-2-[^18^F]-2-deoxy-d-glucose (22) (2-[^18^F]-AFDG), and evaluated it *in vitro* (Fig. 4A).^70^ In line with their working hypothesis, the authors observed intracellular hydrolysis of 2-[^18^F]-AFDG (22) to 2-[^18^F]-FDG (1), and an enhancement in uptake that was “*GLUT independent*”. This study indicates that increasing lipophilicity enables hexokinase activity to be studied independent of GLUT kinetics. The ^18^F-hexose library was further expanded in 2005, when Neal *et al.* reported a d-glucose-based PET tracer with the ^18^F-probe installed at the C6-position to study the physiology and pathology of d-glucose transporters (GLUTs).^71^

Further studies confirmed that 6-[^18^F]-FDG (23) was transported by GLUTs in a process analogous to d-glucose (2).^72^ Collectively, this provided a structural foundation, based on the site of radiofluorination, to investigate two fundamentally different processes: cell entry, and phosphorylation through direct comparison with 2-[^18^F]-FDG (1). It is interesting to note that 6-[^18^F]-FDG (23) was found to accumulate predominantly in the brain liver and heart, which is in contrast to kidney accumulation noted with 2-[^18^F]-FDG (1).^72^ Since 6-[^18^F]-FDG (23) was also insulin responsive, and GLUTs are associated with insulin-stimulated diseases, the Muzic laboratory investigated 6-[^18^F]-FDG (23) as a tracer for d-glucose transport.^73^ An important finding was the increased concentration of radioactivity observed in skeletal muscle in the presence of insulin. The authors have further corroborated this observation by demonstrating that 6-[^18^F]-FDG (23) can be leveraged to measure d-glucose transport *in vivo* in an insulin-resistant rat model.^74^

### 3.2 Monosaccharides imaging bacteria (Fig. 4B)

**Scheme 4:**
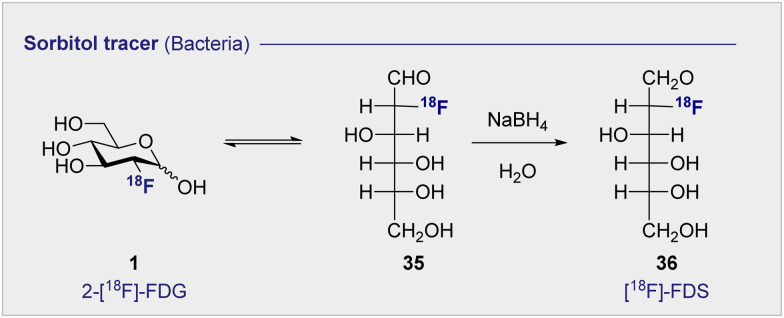
The synthesis of [^18^F]-FDS (36) by reduction of 2-[^18^F]-FDG (1).

Commonly used as a dietary supplement, glucosamine is also one of the core units of peptidoglycans, a polysaccharide that creates the bacterial cell wall from alternating units of *N*-acetylmuramic acid and *N*-acetylglucosamine. As such, glucosamine could be an interesting target for bacterial imaging. In 1990, a glucosamine analogue, *N*-[^18^F]-fluoroacetyl-d-glucosamine (24) ([^18^F]-FAG) was initially investigated for imaging tumours in mice bearing spontaneous hepatomas.^75^ High uptake was observed in the tumour, kidney and liver 60 min post injection (p.i.) with a strikingly highest uptake noted in the tumour at 5.16 ± 0.82% ID per g. Over a decade later, the same compound was evaluated for imaging bacterial infections.^76^ Exploitation of glucosamine's ubiquitous presence in the bacterial cell wall structure could be advantageous in distinguishing between bacterial infection and non-bacterial inflammation. A study in Sprague–Dawley rats, whereby inflammation or infection was induced in a localised manner, showed [^18^F]-FAG (24) selectively imaged bacterial infection over non-bacterial inflammation. By comparison, 2-[^18^F]-FDG (1) was observed to image both infection and inflammation in a manner that it would be difficult to distinguish between the two. By extension, [^18^F]-FAG (24) has become a promising tracer for future development, particularly with regards to differentiating bacterial infection and sterile inflammation. Another potential imaging probe for bacterial infection derives from the reduction of 2-[^18^F]-FDG (1) to generate 2-deoxy-2-[^18^F]-fluorodeoxysorbitol (36) ([^18^F]-FDS) (Scheme 4).

[^18^F]-FDS (36) is metabolised by *Enterobacteriaceae* (Gram-negative bacteria) and not by Gram-positive bacteria or mammalian cells. This difference can be leveraged to enable the targeted imaging of Gram-negative infections. In 2014, a study comparing uptake of [^18^F]-FDS (36) and 2-[^18^F]-FDG (1) in *Escherichia coli* (Gram-negative bacteria), *Staphylococcus aureus* (Gram-positive bacteria) confirmed uptake of 2-[^18^F]-FDG (1) in both strains, however uptake of [^18^F]-FDS (36) was only observed in the *Escherichia coli* cells.^77^ In the same study, *in vivo* experiments in mice injected with both active and inoculated *Escherichia coli* showed [^18^F]-FDS (36) accumulation was exclusive to the area injected with the active infection over the sterile inflammation. The tracer was later translated to human clinical studies which showed no adverse side effects from the injection of the tracer after 24 h as well as good clearance through the bladder.^78^

**Fig. 5:**
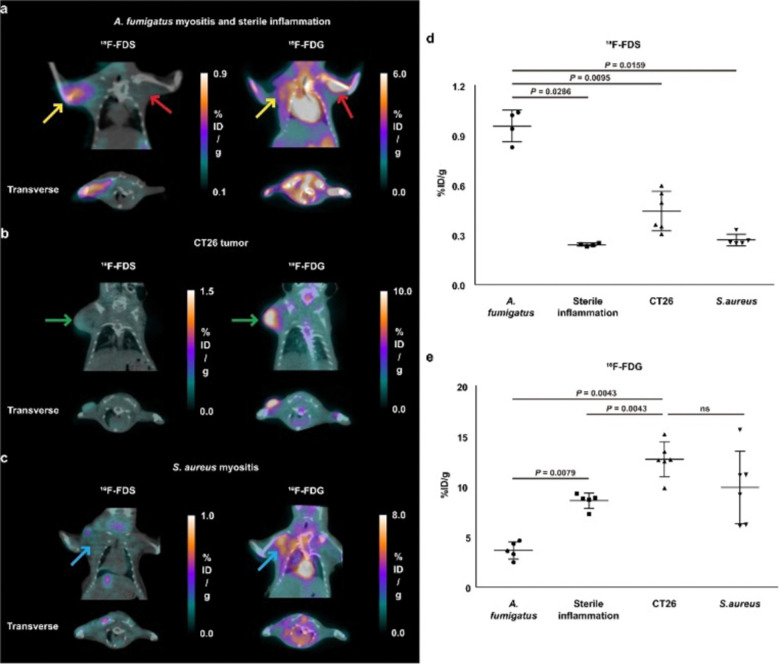
Comparison of [^18^F-FDS (36) and ^18^F-FDG in BALB/c mice. (a) Red arrow: mice injected with sterile inflammation; Yellow arrow: *Aspergillus fumigatus*-infected myositis. (b) Green arrow: CT26 tumours. (c) Blue arrow: *Staphylococcus aureus*-infected myositis. (d) [^18^F]-FDS (36) quantification. (e) 2-[^18^F]-FDG quantification. This research was originally published in Nature Communications: D. Y. Kim, A. Pyo, S. Ji, S. H. You, S. E. Kim, D. Lim, H. Kim, K. H. Lee, S. J. Oh, Y. Jung, U. J. Kim, S. Jeon, S. Y. Kwon, S. R. Kang, H. B. Lee, H. Hyun, S. Y. Kim, K. S. Moon, S. Lee, S. J. Kang and J. J. Min. *In vivo* imaging of invasive aspergillosis with ^18^F-fluorodeoxysorbitol positron emission tomography, *Nat. Commun*., 2022, **13**, 1–11. Reused under the Creative Commons Attribution 4.0 International License (https://creativecommons.org/licenses/by/4.0/).

More recently [^18^F]-FDS (36) research has been expanded to include fungal infections, in particular the *Aspergillus* strain (Fig. 5).^79,80^ Initial comparative studies with *Escherichia coli* showed poor uptake in *Aspergillus fumigatus* by comparison with the Gram-negative bacteria. In addition, [^18^F]-FDS (36) accumulation was outmatched by 2-[^18^F]-FDG (1) (0.290 ± 0.030 and 8.416 ± 0.964% ID mL^−1^ respectively) in infected lungs.^79^ However, later *in vitro* studies indicated comparable [^18^F]-FDS (36) uptake in *Aspergillus fumigatus*, *Rhizopus arrhizus* and *Candida albicans* with *Escherichia coli*.^80^ When translated to an animal model (immunosuppressed BALB/c mice) visualisation of the infected muscle was significant, with an mean infected muscle to normal muscle ratio of 8.90 ± 1.81 at 2 h.

A comparative study with 2-[^18^F]-FDG (1) in BALB/c mice highlighted a 3.9-fold higher [^18^F]-FDS (36) uptake in infected muscle than inflamed tissue (0.95 ± 010% ID per g and 0.24 ± 0.01% ID per g respectively). 2-[^18^F]-FDG (1) on the other hand showed higher uptake in the site of sterile inflammation (Fig. 5). Selective uptake for infected tissue over normal tissue was further replicated in the brain and lung, with as a high as 30.7-fold higher uptake in the infected lung tissue over the normal making it a promising diagnostic tool for fungal infections in the future.

### 3.3 d-Glucose uptake: the GLUT and SGLT pathways (Fig. 4C)

**Fig. 6:**
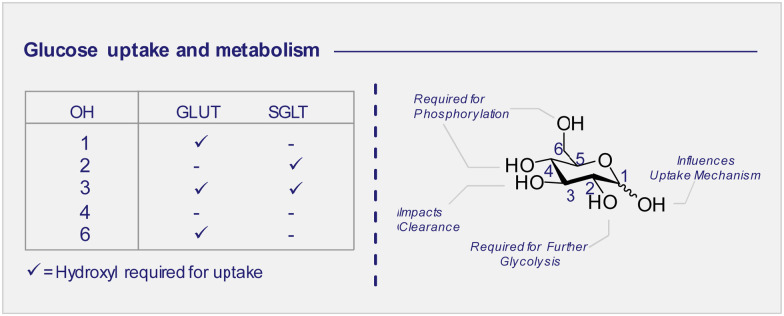
d-Glucose uptake: hydroxyl groups required to enable GLUT or SGLT uptake (left). d-Glucose metabolism: structure activity profile dictating which positions can be modified for tracer design (right).

Whilst 2-[^18^F]-FDG (1) quickly rose to prominence as an indispensable tool for measuring the kinetics of d-glucose metabolism through the GLUT pathway, this particular structural isomer proved unsuitable for exploring sodium d-glucose co-transporter pathways. The SGLT pathway facilitates absorption of d-glucose through the lining of the small intestine mucosa, where an ATPase biases the Na^+^/K^+^ gradient across the proximal tubule cell. This imbalance creates a downhill sodium gradient which can be used by the SLGT proteins to actively transport the d-glucose through the apical membrane.^81–83^ Although 2-fluoro sugars have found application as SGLT2 inhibitors,^84,85^ 2-[^18^F]-FDG (1) is not a substrate for SGLT and as such could not be used to study these proteins *via* PET.^86^ Systematic studies have established that the hydroxyl groups at C1, C3 and C6 of d-glucose are required for GLUT uptake, whilst the C4 position is less critical (Fig. 6). In contrast, the C2 and C3 hydroxyl groups are essential for SLGT transport providing a structural foundation to distinguish between these pathways.^87–89^ 4-[^18^F]-Fluoro-4-deoxy-d-glucose (25) (4-[^18^F]-FDG) has thus been employed to investigate the physiological roles of SGLTs and GLUTs by PET in mice and found to have a high affinity for SGLTs, whilst also being a substrate for GLUT uptake (Fig. 4C).^87,90^ In parallel, the methylated analogue, α-methyl-4-[^18^F]-fluoro-4-deoxy-d-glucopyranoside (26) (αMe-4-[^18^F]-FDG), was designed as a SGLT-specific tracer based on previous studies on the methylated d-glucose analogue (αMDG). αMDG was shown to be selective for SGLT uptake over GLUT,^91^ (a high affinity substrate for SGLT1 and SGLT2 but not GLUTs)^92^ and a poor substrate for hexokinase.^22,86^ In 2016, as part of a wider study of d-glucose homeostasis, the functional expression of SGLTs and GLUTs was measured in mice by comparative analysis of 2-[^18^F]-FDG (1) and αMe-4-[^18^F]-FDG (26). As expected, 2-[^18^F]-FDG (1) produced a signal in the brain, heart and kidneys and was excreted by the bladder.^90^ In stark contrast, the study with αMe-4-[^18^F]-FDG (26) revealed poor brain penetration such that there was no observable signal.

Importantly, 4-[^18^F]-FDG (25) accumulated in the brain but only in regions where SGLTs are expressed. It is also pertinent to note that αMe-4-[^18^F]-FDG (26) is not a substrate for hexokinases and thus it is not accumulated through the GLUT mechanism like 2-[^18^F]-FDG (1).^86^

**Fig. 7:**
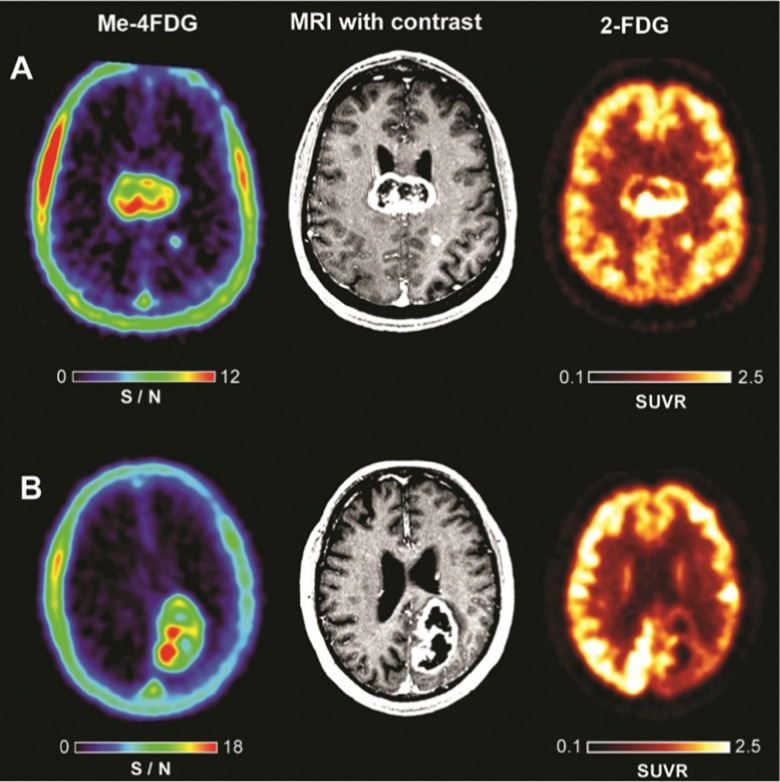
Comparison of PET imagers αMe-4-[^18^F]-FDG (left) and 2-[^18^F]-FDG (right) with MRI (middle) in WHO Grade IV astrocytoma patients. This research was originally published in the Journal of Neuro-oncology: V. Kepe, C. Scafoglio, J. Liu, W. H. Yong, M. Bergsneider, S. C. Huang, J. R. Barrio and E. M. Wright, Positron emission tomography of sodium d-glucose cotransport activity in high grade astrocytomas, *J*. *Neurooncol*., 2018, **138**, 557–569. Reused under the Creative Commons Attribution 4.0 International License (https://creativecommons.org/licenses/by/4.0/).

Recently, Wright and co-workers have validated αMe-4-[^18^F]-FDG (26) as a SGLT specific PET imaging probe in patients with high-grade astrocytoma.^93^ In contrast to 2-[^18^F]-FDG (1), which was broadly distributed in the brain resulting in poor to no resolution, αMe-4-[^18^F]-FDG (26) accumulated in the tumour with a high ratio to the background signal. Indeed, a mass as small as 6 mm in a grade IV tumour patient was observable. And the imaging was sufficiently resolved to be comparable with gadolinium-based MRI (Fig. 7).

### 3.4 d-Mannose-based ^18^F radiotracers (Fig. 4D)

No etiological survey of hexoses in personalised medicine would be complete without a comment on the C2-epimer of 2-[^18^F]-FDG (1): 2-deoxy-2-[^18^F]-fluoro-d-mannose (6) (2-[^18^F]-FDM) (see Scheme 2 and Fig. 4D). A study by Fukada *et al.* evaluated the properties and activity of 2-[^18^F]-FDM (6) as a radiotracer in tumour tissue.^94^ The synthesis of the radiotracer was achieved following the established method for the Direct comparison with 2-[^18^F]-FDG (1) revealed that both radiotracers displayed a similar high uptake in tumour tissue, and high tumour to tissue ratio was observed across most organs. Furthermore, PET studies performed on a rabbit tumour with 2-[^18^F]-FDM (6) showed clear distinctions between the diseased tissues, tumour and lymph node metastases.

The synthesis of 2-[^18^F]-FDM (6) was further optimised through nucleophilic displacement (yield: 50–68%, purity: 97.6–98.7%) in 2013, increasing appeal of 2-[^18^F]-FDM (6) as a potential tumour imaging agent.^95^*In vitro* studies, in AH109A cells, showed 2-[^18^F]-FDM (6) had rapid uptake by tumour cells (30 minutes) and that this uptake was sensitive to co-administration with d-glucose. When studied in AH109A bearing rats, the tumour to muscle ratio was similar to 2-[^18^F]-FDG (1) (5.30 ± 1.54 and 6.20 ± 1.63 respectively) but 2-[^18^F]-FDM (6) had less uptake in the brain. As a result, 2-[^18^F]-FDM (6) could be developed for selective imaging of brain tumours in the future.

2-[^18^F]-FDM (6) has also been investigated for the identification of plaque inflammation to image atherosclerosis.^96,97^ Macrophages involved in this process consume higher levels of d-glucose compared to surrounding tissue within the plaque making this a prime candidate for d-glucose related PET imaging. *In vivo* rabbit studies found uptake of 2-[^18^F]-FDG (1) and 2-[^18^F]-FDM (6) *via*d-glucose transporters in atherosclerotic lesions was comparably high. Furthermore, *in vitro* studies in macrophages showed ∼35% higher uptake of 2-[^18^F]-FDM (6) over 2-[^18^F]-FDG (1) resulting from reduced hexokinase II inhibition. Antibody binding studies to d-mannose receptors expressed by M2 macrophages showed decreased antibody binding with 2-[^18^F]-FDM (6) but not with 2-[^18^F]-FDG (1). This preferred binding of 2-[^18^F]-FDM (6) could be harnessed in the future to detect progressive inflammation.

Another comparative study of 2-[^18^F]-FDG (1) and 2-[^18^F]-FDM (6) investigated specific labelling of the more commonly associated T lymphocyte-activating antigen (CD80) with two pyrazolocinnoline-based radiotracers in a mouse model. High accumulation of 2-[^18^F]-FDG (1) and 2-[^18^F]-FDM (6) in monocytes and macrophages that are present in atherosclerotic plaque was observed through active transport using GLUTs. CD80 was found to be essentially involved in the inflammatory processes of atherosclerosis and therefore, could be useful as a marker in early diagnosis.^97^

### 3.5 d-Galactose-based ^18^F radiotracers (Fig. 4E)

**Scheme 5:**
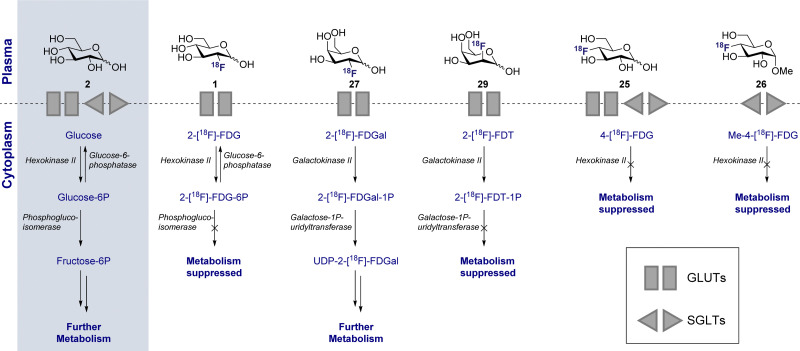
Metabolism of various ^18^F radiotracers in comparison to d-glucose (2).

2-Deoxy-2-[^18^F]-fluoro-d-galactose (27) (2-[^18^F]-FDGal), the C4-epimer of 2-[^18^F]-FDG (1) (Fig. 4E), was rigorously investigated in the late 1980s to elucidate d-galactose metabolism pathways.^98^ The parent monosaccharide, d-galactose, is a core building block in glycoprotein synthesis *via* phosphorylation, at the C1 position, to d-galactose-1-phosphate (Gal-1-P), and also serves as a central energy source through subsequent conversion of Gal-1-P to d-glucose *via* galactose-1-phosphate uridyltransferase and UDP galactose-4′-epimerase (Scheme 5).^99^ Although the galactokinases responsible for phosphorylation are prevalent throughout the body, they are most prolific in the liver. This reflects the importance of efficient d-galactose excretion in human health and its diagnostic importance. Initial biodistribution studies of 2-[^18^F]-FDGal (27) in rats highlighted a high liver to blood ratio, and this was confirmed in a second study in rabbits. An important finding of this study was the observation that subsequent administration of d-galactose decreased the levels of tracer in the liver. However, this effect was not observed after d-glucose (2) administration, indicating that uptake occurs through a competitive process with d-galactose (16) but not d-glucose (2). The authors also observed a correlation between 2-[^18^F]-FDGal (27) uptake and organospecific galactokinase activity, which followed the trend: liver > kidney > brain > muscle. This further supports the notion that uptake occurs through the d-galactose mechanism. Having established the mode of uptake, two main metabolites of 2-[^18^F]-FDGal (27) were identified. The phosphorylation product, 2-deoxy-2-[^18^F]-fluoro-d-galactose-1-phosphate, was formed immediately by galactokinase and was responsible for 81% of ^18^F tracer activity. In addition, ^18^F-FDGal-1-P, was further uridylated to UDP-[^18^F]-FDGal by UDP-glucose. It is noteworthy that these metabolites were also identified in the heart, lung, spleen and small intestine.^100^

The d-galactose selectivity observed for liver tissue presented an array of opportunities for clinical translation and in 1989 a study reporting the application of 2-[^18^F]-FDGal (27) as a radiotracer for liver tumour imaging was reported.^101^ Interestingly, both metabolites (*vide supra*) were identified in liver tumours in mice (mammary carcinoma) and rats (Yoshida carcinoma). The efficiency of 2-[^18^F]-FDGal (27) in well differentiated hepatomas in mice and rats was also investigated leading to the conclusion that higher uptake in well-differentiated hepatomas (spontaneous hepatoma C3H mice) occurs than in hepatomas that were less differentiated (*e.g.* MH129P). From the perspective of tumour imaging, it is pertinent to highlight that 2-[^18^F]-FDGal (27) has been reported to outperform 2-[^18^F]-FDG (1), with tumour to tissue ratios of 13.7 and 6.46, respectively, having been noted in poorly differentiated hepatoma.^94,99^

In 2008, a report by Sørensen *et al.* quantified d-galactose (16) uptake and liver metabolism using 2-[^18^F]-FDGal (27). Ten pigs were anesthetised, injected with 2-[^18^F]-FDGal (27) and monitored by PET. The hepatic clearance rate of 2-[^18^F]-FDGal (27) was found to be 600 μmol min^−1^ L^−1^ in tissue, which is in-line with previous studies, underscoring the potential of 2-[^18^F]-FDGal (27) as an accurate, non-invasive indicator of general liver health.^102^ In 2011, the same group reported a study of 2-[^18^F]-FDGal (27) in 39 patients having either known hepatomas or suspected liver cancer was conducted.^103^ The patients were administered with 2-[^18^F]-FDGal (27) intravenously and tumours were detected in 22 out of 23 patients with active liver cancer. Identical detection rates were noted with multiphase contrast-enhanced CT (ceCT). 2-[^18^F]-FDGal (27) has also been employed to determine dose-response relationships for Stereotactic Body Radiation Therapy (SBRT) to study liver metabolism (as well as recovery) in more detail.^104^ Furthermore 2-[^18^F]-FDGal (27) has been successfully leveraged to measure liver function *in vivo* as an non-invasive alternative to arterial blood sampling.^105^

A structural isomer of 2-[^18^F]-FDGal (27), 6-deoxy-6-[^18^F]fluoro-d-galactose (28) (6-[^18^F]-FDGal) was also investigated for imaging d-galactose (16) metabolism.^106^ Moving the ^18^F-radiolabel to the C6-position instead of the C2-position, retained rapid biodistribution from the blood to the remaining tissues and organs. However, rapid excretion also ensued with 56% of the radiotracer found in the urine at 60 min p.i.

The C6–OH in d-galactose has previously been shown as a requirement for galactokinase binding. This was evident in the metabolites that were detected as only very low levels of 6-[^18^F]-FDGal-1P were detected in the liver in comparison to the non-galactokinase dependant oxidised metabolite galactonate, potentially aiding in rapid excretion. Furthermore, this tracer showed no competitive uptake with d-galactose. This, combined with the previous experiments determined the C6-OH is preferably left unsubstituted and as such the tracer was not further investigated.

### 3.6 d-Talose-based ^18^F radiotracers (Fig. 4F)

2-Deoxy-2-[^18^F]-fluoro-d-talose (29) (2-[^18^F]-FDT) was synthesised by Diksic and Jolly in 1985 by an aqueous fluorination protocol.^107^ Since this species is formally a C2 and C4 epimer of d-galactose and d-mannose, respectively, it is an attractive small molecule for the study of galactokinase activity (Fig. 4F and Scheme 5). Indeed, an earlier study by Alvarado demonstrated that d-talose was phosphorylated by galactokinases in *Saccharomyces fragilis*.^108^

Biodistribution studies performed from the early 90s in tumour bearing mice (fibrosarcoma) revealed that the highest uptake occurred in the liver (34.9% dose per g), followed by kidney (15.9% dose per g) and small intestine (12.9% dose per g). However, 2-[^18^F]-FDT (29) was also characterised by high liver uptake in normal rats. This was inhibited by parallel administration of d-galactose, supporting the notion that a d-galactose uptake mechanism is operational, much like 2-[^18^F]-FDGal (27).^109^ These promising results were further expanded upon two years later by the same group.^110^ Further NMR spectroscopic analysis determined that 2-[^18^F]-FDT-1-phosphate was the only metabolite generated in tumour-bearing mice (administration 60 mg kg^−1^). Furthermore, *in vitro* analysis revealed that 2-[^18^F]-FDT (29) is rapidly phosphorylated to 2-[^18^F]-FDT-1-phosphate by galactokinase, but subsequent uridylation does not occur. This indicates that the subtle C2 epimerisation (d-galactose *versus*d-talose), renders 2-[^18^F]-FDT (29) an unsuitable substrate for galactose-1-phosphate-uridyltransferase (Scheme 5). 2-[^18^F]-FDT-1-phosphate is thereby trapped in tumour tissue, ensuring accumulation for PET. Despite its appeal as a tracer for d-galactose metabolism, particularly as the lack of further metabolism simplifies data analysis, 2-[^18^F]-FDT (29) has been comparatively under-explored since the 1990s.

### 3.7 l-Fucose-based ^18^F radiotracers (Fig. 4G)

In stark contrast to the epimeric PET tracers described Fig. 4C–F, l-fucose is a deoxysugar and that differs structurally on account of the deletion at C6 position and its inverted absolute configuration (l- *versus*d-). In mammalian systems, l-fucose is a ubiquitous component of *N*-linked glycans where it is frequently linked to the reducing end β-*N*-acetylglucosamine in glycolipids.^111^ Given the prevalence of glycolipids in the plasma membrane, they are important tumour biomarkers for cell growth, division, antigenicity and differentiation. Consequently, l-fucose-based PET analogues hold great promise in cancer diagnostic and personalised medicine.

In 1990, Ishiwata *et al.* examined 6-[^18^F]-fluoro-l-fucose (30) (6-[^18^F]-FFuc) as an avenue for monitoring glycoconjugate synthesis in tumours (Fig. 4G).^112^*In vivo* studies in FM3A-bearing mice revealed that the main metabolite was GDP-6-[^18^F]-FFuc, thereby implicating guanidylation by GDP-fucose-pyro-phosphorylase is being rate determining in metabolism. Upon co-administration of l-fucose, uptake of 6-[^18^F]-FFuc (30) was significantly impeded thereby indicating competition for the l-fucose uptake mechanism. Two years later, in a broader study of several sugar-based tracers across five different tumour models, 6-[^18^F]-FFuc (30) was found to have a much poorer uptake by tumour-bearing rats and mice than 2-[^18^F]-FDG (1).^113^ Although uptake was comparatively low, the tumour to tissue ratio in the brain was far superior at 7.27 ± 0.77 compared with 1.28 ± 0.45 for 2-[^18^F]-FDG (1). Moreover, 2-[^18^F]-FdFuc (31) was also found to have a slightly improved uptake than its counterpart 6-[^18^F]-FFuc (30), however showed lower tumour to tissue ratio in the brain at 2.88 ± 0.48.

### 3.8 Sialic acid-based ^18^F radiotracers (Fig. 4H)

Sialic acids are mostly found at the termini of sugar chains and as such play an exceptional role in nature. Coating the cell surface, they control a wide range of biochemical processed *e.g.* cell recognition. Inspired by the prevalence of sialic acids in regulatory gangliosides on the plasma membrane, Ishiwata *et al.* prepared two ^18^F-*N*-acetyl-neuramic acid tracers, *N*-acetyl-3-[^18^F]-fluorosialic acid (32 and 33) (3-[^18^F]Neu5Ac-C3_ax._ and C3_eq._) and *N*-acetyl-2-deoxy-2,3-difluorosialic acid (34) (2,3-di[^18^F]Neu5Ac), to investigate glycoconjugate metabolism of tumours.^114^ However, selective uptake could not be established across the range of tumour models that were tested in addition to poor overall uptake and high clearance rates. Even though sialic acids have shown little promise in PET, alternative methods *e.g.* Bertozzi's modified Staudinger reaction, for *in vivo* imaging of sialic acid surfaces have been developed.^115^ Sialic acids are not only important for tumour progression, but also decorate the end of glycolipids *e.g.* gangliosides, and serve important regulatory purposes. For more information on gangliosides, please see Section 6, ^18^F-Modified Ganglioside Tracers.

## 4. ^18^F-modified disaccharide tracers

### 4.1 Lactose-based ^18^F radiotracers (Fig. 8A)

The natural abundance of simple disaccharides, coupled with the modular nature of carbohydrates in a broader sense, manifests itself in the next level of PET tracer structural complexity. Lactose results from the union of d-glucose (2) and d-galactose (16) through a characteristic β-(1 ± 4) glycosidic bond. Given the independent success of the constituent monosaccharides in nuclear medicine, 2′-[^18^F]-FDL (37) has been synthesised from 2-[^18^F]-FDG (1) *via* enzymatic catalysis leveraging galactosyl transferase.^116^ Specifically designed to visualise expression of the LacZ gene, the tracer unfortunately did not accumulate in any of the organs in normal mice and was rapidly excreted through the bladder.

**Fig. 8:**
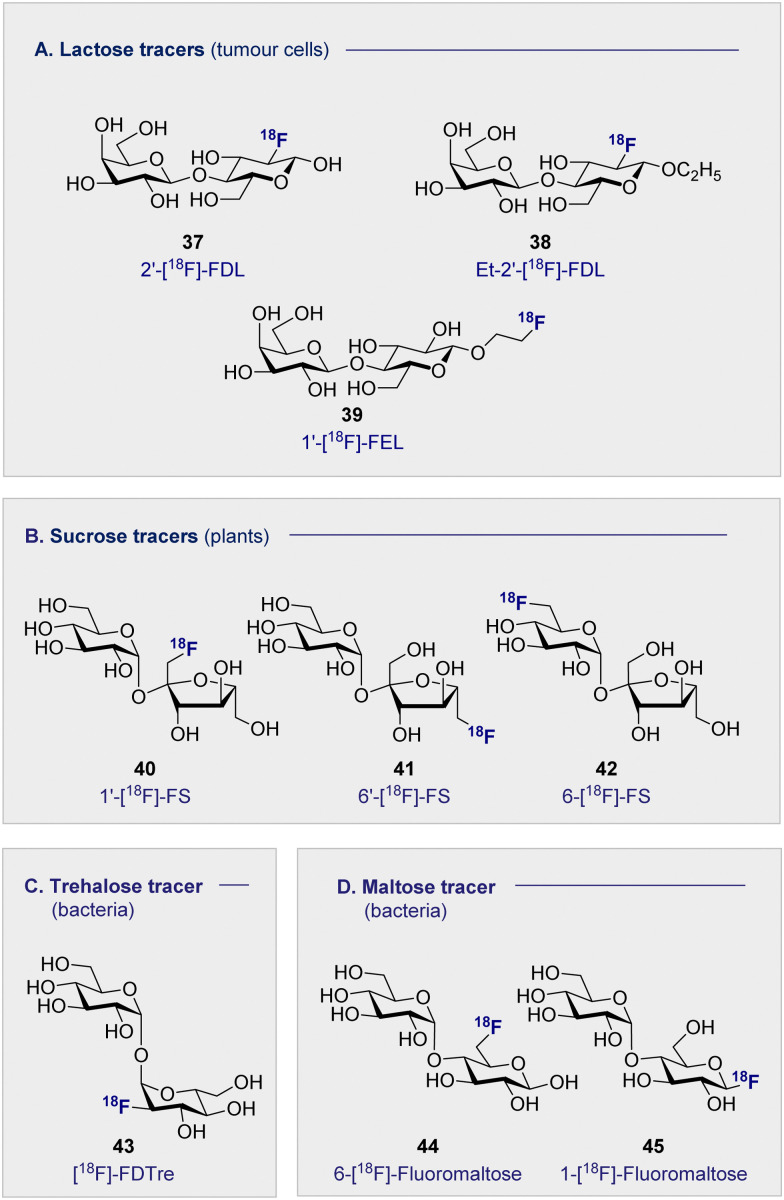
Structures of disaccharide-based ^18^F radiotracers.

The hepatocarcinoma-intestine-pancreas/pancreatitis-associated protein (HIP/PAP) is overexpressed in peritumoral pancreas and has previously shown to have a high affinity for d-lactose. Consequently, ethyl-2′-deoxy-2′-[^18^F]-fluorolactose (38) (Et-2′-[^18^F]-FDL) was later developed to facilitate imaging of pancreatic carcinoma and was found to bind specifically to peritumoral pancreatic tissue in mice (Fig. 8A).^117^ A notable advance stemmed from the introduction of 1′-[^18^F]-FEL (39), a tracer with potentially similar activity to Et-2′-[^18^F]-FDL (38) but which could be accessed much more efficiently.^118,119^ In tumour-bearing mice, 1′-[^18^F]-FEL (39) showed a significant increase of uptake in the peritumoral tissue (1.29 ± 0.295% ID per g), in comparison with the wild type (0.090 ± 0.010% ID per g). It is pertinent to note that uptake was determined by autoradiography rather than by the PET image due to poor resolution.^120^ The uptake in the surrounding tissues, no significant difference was noted compared to the control mice: this is consistent with the previous discussion on the lactose-based tracer Et-2′-[^18^F]-FDL (38). Interestingly, 1′-[^18^F]-FEL (39) was shown to be selective for the peritumoral tissue and no tracer accumulation in the pancreas was observed in the control group mice.

### 4.2 Sucrose-based ^18^F radiotracers (Fig. 8B)

Whilst ^18^F-modified carbohydrates have a venerable history in studying mammals and bacteria, ^18^F PET is also a highly effective paradigm for the study of plant metabolism. Capitalising on the formation of sucrose as the ultimate product of photosynthesis, ^18^F-labelled derivatives have been instrumental in this endeavour. In 2012, the Fowler group prepared 1′-[^18^F]-fluoro-1′-deoxysucrose (40) (1′-[^18^F]-FS) through a concise sequence involving enzymatic synthesis of the triflate precursor with concomitant radiofluorination with K^18^F.^121^

This was followed by chemical syntheses of 6′-deoxy-6′[^18^F]-fluorosucrose (41) (6′-[^18^F]-FS) and 6-deoxy-6[^18^F]-fluorosucrose (42) (6-[^18^F]-FS).^122^ All three derivatives were successfully translocated confirming that OH to ^18^F bioisosterism does not compromise membrane transport and underscores the utility of PET in the study of plant physiology and biochemistry.^123^

### 4.3 Trehalose-based ^18^F radiotracers (Fig. 8C)

The persistent threat presented by antibiotic resistance has led The World Health Organisation to classify it as one of the top threats to human health.^124^ If current projections persist, bacterial resistance will surpass cancer as the leading cause of human deaths worldwide by 2050. This critical situation continues to be exacerbated by the unnecessary administration of antibiotics, often in scenarios in which a bacterial infection has not been confirmed. The development of selective diagnostic procedures to facilitate rapid, early detection is set to play a central role in alleviating this current crisis. Since clinical success is conditional on the ability to target pathogenic bacterial cells in the presence of eukaryotic cells, simple ^18^F-modified oligomers have been intensively investigated to mitigate the lack of selectivity associated with 2-[^18^F]-FDG.

Trehalose, a disaccharide comprised of two d-glucose subunits linked by a α-(1 → 1) glycosidic bond, is readily uptaken by *Mycobacterium tuberculosis*; a process that has been studied by a FITC-labelled probe,^125^ and leveraged for PET through the introduction of an ^18^F-lablelled species.^126^ This latter study revealed selective uptake of ^19^FDTre analogues by SugABC-LpqY, which is a trehalose transporter in *Mycobacterium smegmatis* cells. Subsequent comparative studies in mammalian and bacterial cell have further cemented trehalose as a promising future tracer core, in particular [^18^F]-FDTre (43) tracer uptake shown at a picomolar level.^127^ The suppression of uptake in *Mycobacterium smegmatis* when ^19^F-trehalose is introduced further supports a trehalose-based uptake mechanism.

### 4.4 Maltose-based ^18^F radiotracers (Fig. 8D)

Targeted diagnosis of bacterial infection can be further expanded to include the maltose-based tracers. In particular, the exclusivity of the maltodextrin transport complex present in bacteria renders maltose,^128–130^ and its higher oligomers, an attractive candidate for selective tracer development.^131,132^ In 2014, Gambhir and co-workers reported the synthesis of 6-[^18^F]-maltose (44) as an imaging probe and evaluated it *in vitro*.^133^ When co-administered with maltose, uptake was supressed in *Escherichia coli*, suggesting the involvement of a common uptake pathway. Furthermore, the authors noted that whilst 6-[^18^F]-maltose (44) is sequestered by various bacteria, both Gram-positive and Gram-negative (*Escherichia coli* ATCC 33456, *Pseudomonas. aeruginosa* and *Listeria monocytogenes*), uptake by mammalian cancer cells (human breast cancer, human cervical cancer) was not observed. A murine model revealed that 6-[^18^F]-maltose (44) accumulated in muscles that were infected with bacterial myositis but not in inflamed tissue: this is noteworthy given the clinical relevance of distinguishing between infection and inflammation.^134,135^ However, the significant uptake observed in the blood and the slow clearance from major organs, *i.e.* brain, liver and kidneys, compromised image resolution. The same group have trialled 1-[^18^F]-fluoromaltose (44), but *in vivo* instability observed in the uptake experiments resulted in defluorination and subsequent incorporation of the fluoride ion into the bone.^136^

## 5. ^18^F-modified oligosaccharide tracers

Of the naturally occurring oligosaccharides that have been considered for diagnostic applications using PET, maltodextrins have emerged as being particularly well-suited to the study of bacterial infections. Like their lower d-maltose (46) analogues, these polysaccharides are comprised of d-glucose (2) monosaccharides which are connected by α-(1 → 4) glycosidic linkages. Their highly hydrophilic nature renders them impermeable to the cell membrane, thereby requiring the involvement of maltodextrin transporter proteins to facilitate cellular uptake.^137^ In the case of ^18^F-radio-labelled derivatives, this physicochemical signature ensures that the target to background ratio is enhanced as residual tracer that is not taken into the bacterial cells is cleared rapidly and efficiently. The striking breadth of clinical specificity that can be realised by increasing molecular weight across the d-glucose (2), d-maltose (46), maltodextrin series serves to highlight unlocking the potential of seemingly simple sugars for their use in personalised medicine.

A report by Murthy and co-workers in 2011 described the application of a maltodextrin-based fluorescent imaging probe to detect bacteria *in vivo* with high sensitivity and specificity.^138^ In this study, the authors report that maltodextrin transporters tolerate anomeric substitutions, suggesting that the reported label can be installed at the reducing end of the oligomer. As a proof of concept upon which to advance a PET tracer, two target compounds based on maltohexaose (47) were prepared with a fluorescent dye introduced by click chemistry.

**Fig. 9:**
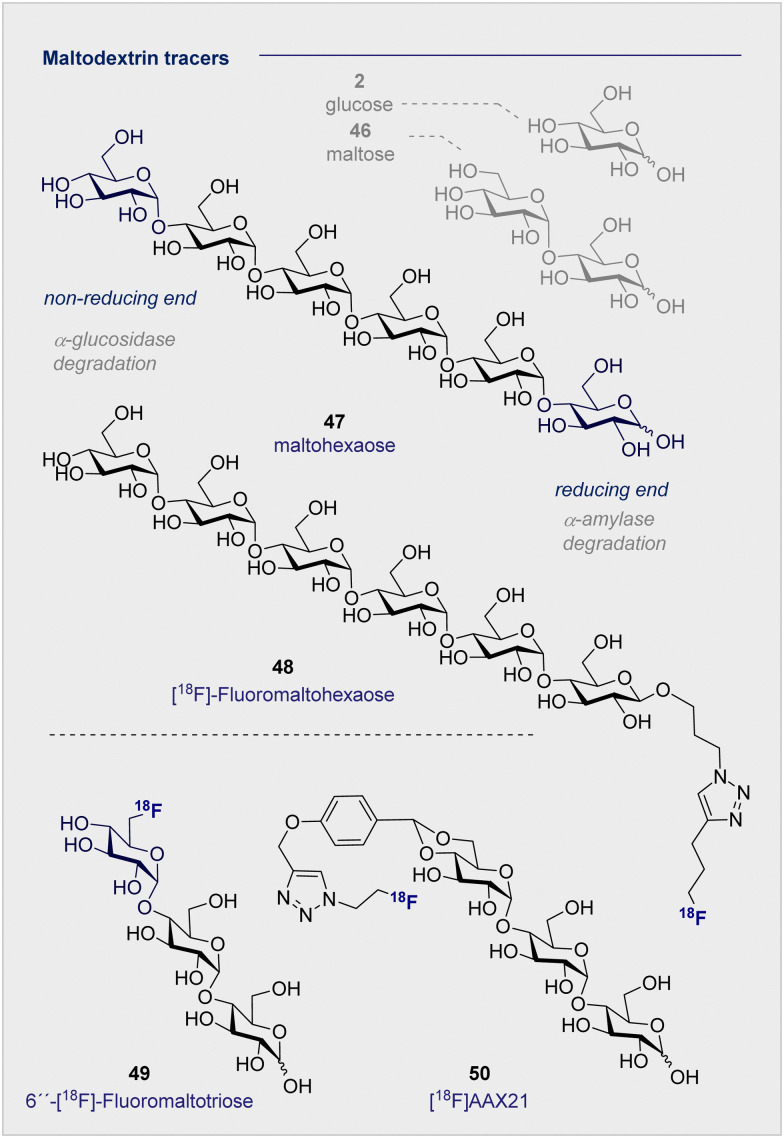
Selected maltodextrin-based ^18^F-radiotracers.

Following incubation with Gram-positive and Gram-negative bacteria, accumulation in the millimolar range was noted in both cases. The initial findings were further corroborated by *in vivo* experiments in rats infected with *E. coli*. These findings based on the fluorescent probes provided a foundation for the subsequent advancement of a maltohexaose ^18^F-radio tracer for PET imaging (Fig. 9).^139^ Substituting the terminal fluorescent dye with an ^18^F motif, again *via* click chemistry, was performed. The authors observed high accumulation in bacterial cells during *in vivo* studies with *Escherichia coli* in rats, with [^18^F]- maltohexaose (48) being selectively metabolised by bacteria, relative to 2-[^18^F]-FDG (1). Building on this success, a second-generation PET tracer, 6′′-[^18^F]-fluoromaltotriose (49), was introduced in 2017 which is based on the shorter oligomer maltotriose.^140^ The introduction of 6′′-[^18^F]-fluoromaltotriose (49) led to improvements in both the pharmacokinetics and target-to-background ratios relative to 6-[^18^F]-maltose (44). It is interesting to note that 6′′-[^18^F]-maltotriose (49) was less strongly absorbed into the blood and muscles, and that it showed a longer residence time in *Escherichia coli*, likely due to a slower metabolism of digesting maltotriose into d-glucose units. There are, however, several limitations that are worthy of consideration. Maltodextrin-based PET tracers are only suitable to detect bacteria that are located extracellular, whereas intercellular scenarios such as *Mycobacterium tuberculosis* present a challenge.

Furthermore, the maltodextrin transport system is complex and can differ depending on the bacterial species (Gram-negative/positive).^141^ Finally, enhancing serum stability is a core objective to mitigate tracer degradation in the blood: efficient hydrolysis by α-glucosidase (non-reducing end) and α-amylase (reducing end) is a persistent threat when utilising maltohexaoses that must be alleviated. To address these issues, Faust, Gilmour and co-workers initiated a study of maltose-based ^18^F radiotracers of varying chain length (1–6 d-glucose units) to study their stability towards α-amylase in blood.^10,142^ An initial control experiment with a model maltodextrin substituted at the anomeric position confirmed degradation by α-glucosidase (at the non-reducing end) in blood. Following independent incubation of each ^18^F-PET tracer in human blood serum, [^18^F]-maltopentaose and [^18^F]-maltohexaose were also found to undergo rapid degradation, whereas [^18^F]-maltotriose (50) ([^18^F]AAX21) showed promising stability (over 120 min, amylase activity 51 U L^−1^). Furthermore, incubation of the (50) [^18^F]AAX21 in murine serum, which has a 100-fold higher α-amylase activity, confirmed the stability of the tracer towards enzymatic hydrolysis. This indicates that the position of the label provides a structural handle by which to augment stability *in vivo* and, that for bacterial uptake, a free anomeric center is required on the terminal monosaccharide to access the cytoplasm through MalFGK_2_.^128,130,143^

## 6. ^18^F-Modified ganglioside tracers

**Fig. 10:**
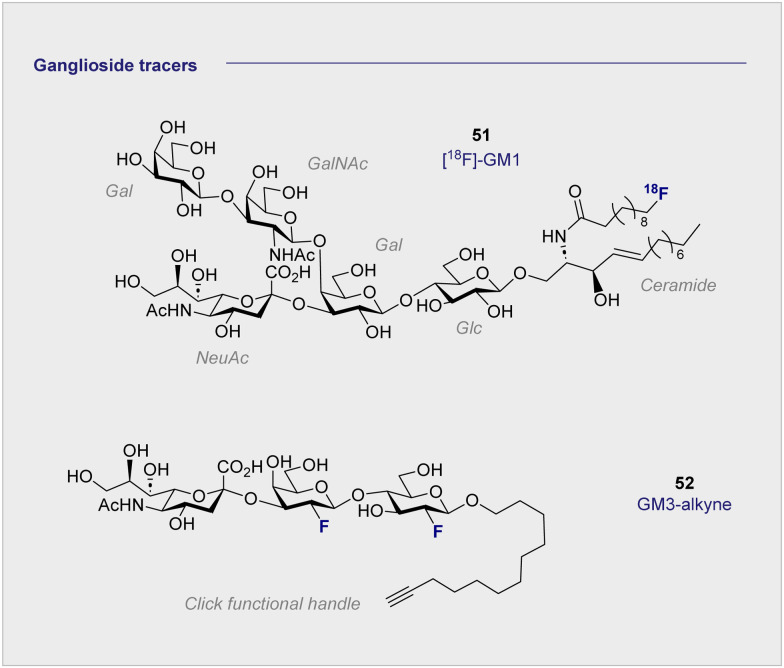
Structures of modified gangliosides GM_1_ (upper) and GM_3_ (lower).

The monosialyl ganglioside GM1 is highly localised in the brain, specifically in the postsynaptic neuronal membranes, which renders it attractive in the study of neurodegenerative disorders.^144,145^ In 2020, the Schou laboratory prepared an ^18^F-radiolabelled analogue of GM1, [^18^F]-GM1 (51) for study in the non-human primate brain (Fig. 10).^146^ However, upon injection, a biodistribution study in a cynomolgus monkey essentially failed to identify a signal in the brain. The blood to brain ratio was found to be 0.03, indicating that the radioactivity recorded in the region could be attributed to the blood. This observation was further corroborated by an increased signal observed in the vessels peripheral to the blood brain barrier. The study also noted that high levels of [^18^F]-GM1 (51) accumulated in the heart. Given that elevated levels of GM1 have been linked to heart failure, this may compromise further development of the tracer. It is pertinent to note that monosialyl gangliosides GM3 and GM4 are also implicated in a plethora of neurodegenerative processes and constitute structurally simpler candidates for tracer development. A recent synthesis and pre-clinical-evaluation of a GM3-alkyne (52) has shown that the fluorinated epitope had no significant impact on oligodendrocyte viability, whereas the native epitope decreased viability in a dose dependent manner.^147^ It is noteworthy that single site fluorination of the GM4 ganglioside epitope (F *versus* OH) led to an upregulation of oligodendrocyte differentiation: this underscores the modular nature of (fluorinated) carbohydrates and the opportunities that this presents in regulating biological function.^148^

## 7. ^18^F-PET tracers to detect hypoxia

The principle uptake mechanisms associated with carbohydrate-based PET tracer design (*e.g.* 2-[^18^F]-FDG (1)) traditionally centre on d-glucose metabolism pathways: these include active transport *via* GLUTs and/or SGLTs (see Section 3.3).

The clinical translation of candidates that leverage this strategy has proven to be highly successful, but the ubiquity of d-glucose metabolism in cellular metabolism frequently compromises specificity. This consideration becomes particularly acute when attempting to expand the repertoire of PET imaging agents to detect hypoxic tissue (deprivation of oxygen at the tissue level). In tumours, increased hypoxia often results from mutated structures being unable to fully oxygenate the affected cells.^149^ Hypoxia has thus been associated with increased local tumour aggression, resistance to chemotherapy,^150,151^ and probability of metastasis.^152,153^ These hostile conditions promote the survival of malignant cells over healthy cells and inhibit the production of radical oxygen, which is an essential component of effective radiotherapy.^154^ As such, the effective detection of hypoxia through PET-based imaging paradigms is essential in preliminary tumour prognosis. Varying the uptake method to enhance specificity for the target cells would therefore be highly advantageous for PET imaging in hypoxic environments.

### 7.1 ^18^F FMISO – an alternative uptake mechanism

**Scheme 6:**
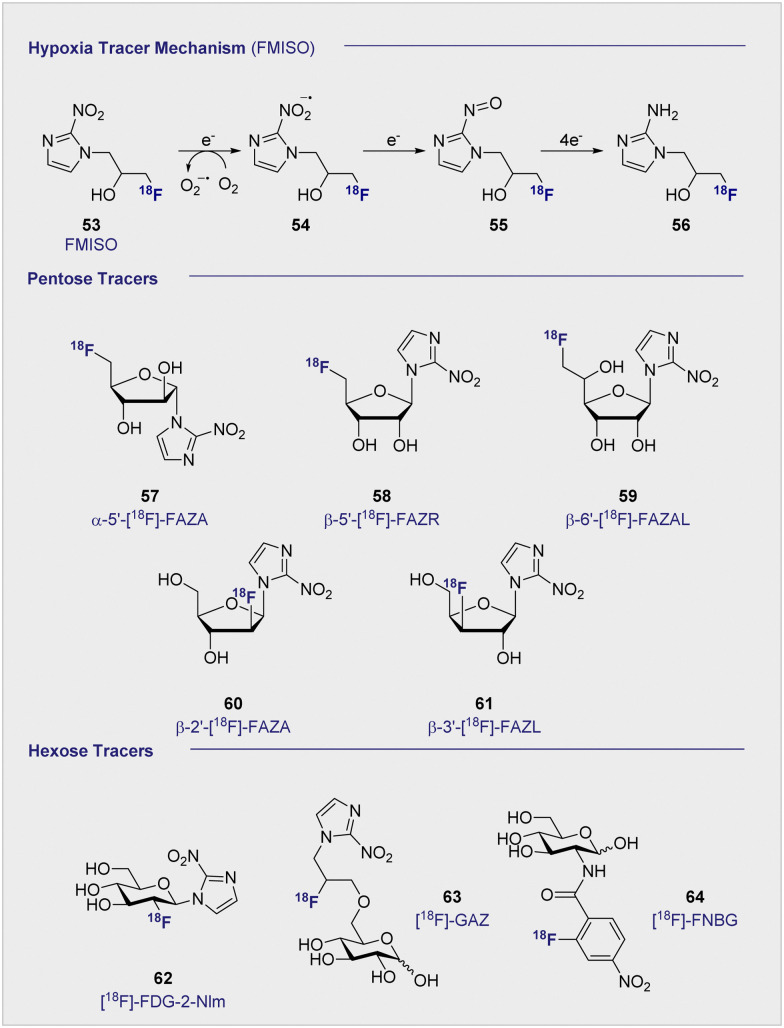
(upper) Proposed, reductive mechanism, post-uptake, through passive diffusion. Oxygenated environment: the FMISO radical anion (54) is restored to its original structure through superoxide radical formation. Absence of oxygen: FMISO radical anion (54) is further reduced to amine (56) that can covalently bind macromolecules, resulting in accumulation of FMISO in the cell. (middle) Structures of selected, pentose-based hypoxia radiotracers (57–61). (lower) Structures of selected hexose-based hypoxia radiotracers (62–64).

Uptake of the small-molecule PET tracer FMISO (53) occurs through passive diffusion (Scheme 6, upper). Once in the cell, the nitro group is sequentially reduced to the amine moiety thus providing a handle for bioconjugation (*e.g.* with *e.g.* glutathione): this ultimately facilitates intracellular bioaccumulation.^155^ It is proposed that this mechanism is only effective in hypoxic conditions, since the presence of oxygen would lead the anionic radical intermediate to be converted back to the original FMISO (53) *via* superoxide radical formation.^156^ Whilst details regarding the exact mechanistic pathway require clarification, the characteristics intrinsic to FMISO (53) render it ideally suited to the study of tumour-associated hypoxic tissue. In the clinic, FMISO (53) has been extensively studied as a hypoxia imaging probe across multiple tumour models, where it has shown to be particularly effective in head and neck tumours as well as breast cancer and gliomas.^157–159^ However, the success and broad utility of FMISO-imaging is not without limitations, most notably poor tumour to background ratio. The paradigm is heavily disadvantaged by the relatively short biological half-life of 50 min, whereas PET tracing regions of hypoxia benefit from longer half-lives to improve image resolution. Longer half-lives facilitate increased uptake by the hypoxic regions whilst allowing clearance from the background tissues. To combat this, the arabinose-derived PET tracer α-5′-[^18^F]-FAZA (57), a carbohydrate derivative of FMISO (53), was developed.^160,161^ In contrast to FMISO (53), α-5′-[^18^F]-FAZA (57) is more hydrophilic thereby enabling the target to be reached faster, whilst being readily expelled through the urinary tract.

**Fig. 11:**
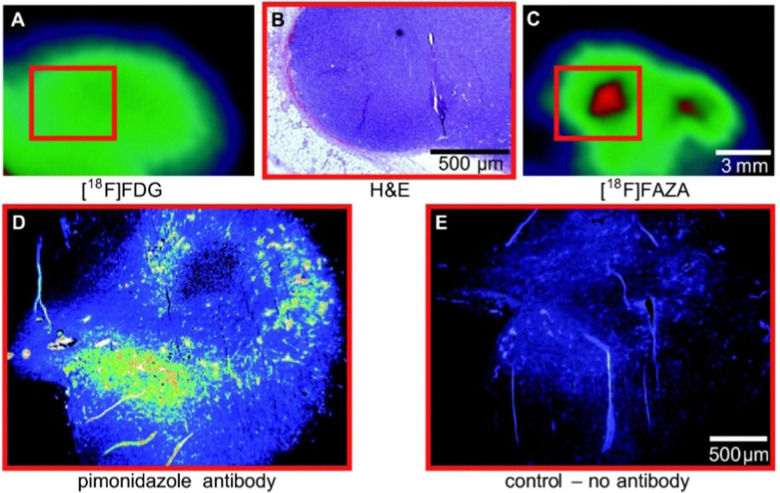
Comparison of 2-[^18^F]-FDG (1) A and [^18^F]-FAZA (57) C on the same CT26 colon carcinoma. B The same CT26 colon carcinoma analyses *ex vivo via* H&E staining of tumor slices (region shown as red square in A and C). D and E Pimonidazole immunohistochemistry *ex vivo*. This research was originally published in Radiation Oncology: F. C. Maier, M. Kneilling, G. Reischl, F. Cay, D. Bukala, A. Schmid, M. S. Judenhofer, M. Röcken, H. J. Machulla and B. J. Pichler, Significant impact of different oxygen breathing conditions on noninvasive *in vivo* tumor-hypoxia imaging using [^18^F]-fluoro-azomycinarabino-furanoside ([^18^F]FAZA), *Radiat. Oncol.*, 2011, **6**, 165. Reused under the Creative Commons Attribution License (https://creativecommons.org/licenses/by/2.0).

Although α-5′-[^18^F]-FAZA (57) has not yet been approved by the FDA, it has shown great promise in animal and patient studies (Fig. 11).^162,163^ One highly pertinent study investigated the effectiveness of α-5′-[^18^F]-FAZA (57) on a cohort of 50 patients with solid tumours that included high-grade glioblastomas, small cell lung carcinoma and malignant lymphoma.^164^ For patients with high-grade glioblastoma, the tumour to background ratio was as high as 15.6 with an average of 5.3 ± 4.7 across 7 patients, partly attributed to poor uptake in normal brain tissue. This improvement in resolution relative to its predecessor subsequently prompted the development of a series of 2-nitroimidazole derivatives in attempt to further enhance cellular uptake. The beta derivative β-5′-[^18^F]-FAZR (58) was evaluated for utilising uptake pathways across the cell membrane to improve efficiency over its predecessor.^165^ Binding experiments, to study uptake mechanism, with nucleoside transporters hENT1/2 and hCNT1/2/3 were carried out to study if β-5′-[^18^F]-FAZR (58) could act as an inhibitor of uridine uptake. Inhibition of all but hCNT3, showed IC_50_ >500 μM. hCNT3 inhibition was shown to be comparable, though almost 2-fold weaker, to the thymidine control (IC_50_ = 65 ± 4 μM and 35 ± 5 μM respectively) indicating β-5′-[^18^F]-FAZR (58) may utilise uptake *via* the hCNT3 transporter.

Another derivative of α-5′-[^18^F]-FAZA (57), β-6′-[^18^F]-FAZAL (59), was specifically introduced by Wanek and co-workers to leverage SLC2A transporters. In particular, SLC29A1, was found to be effective in transporting β-6′-[^18^F]-FAZAL (59) both in and out of the cell, thereby improving tumour to background ratio. This is grounded in an amplified uptake of the tracer, which facilitates accumulation, and increased excretion which lowers the background signal.^166^ A competition study under normoxic conditions revealed inhibition of 2-[^18^F]-FDG (1) uptake, which suggests an interaction with the d-glucose transporter systems: very little β-6′-[^18^F]-FAZAL (59) was retained in the tumours of Wistar rats (Walker 256 mammary carcinosarcoma) by comparison. As a result, no further studies under hypoxic conditions were conducted. β-2′-[^18^F]-FAZA (60), and β-3′-[^18^F]-FAZL (61) have also been prepared but, to the best of our knowledge, details regarding *in vivo* or *in vitro* testing have not been disclosed.^167^ In 2002, Patt and co-workers synthesised and evaluated a nitroimadazole FDG derivative, [^18^F]-FDG-2-NIm (62) to improve on [^18^F]-FMISO's slow kinetics.^168^*In vivo* studies in Wistar rats bearing Walker 256 rat mammary carcinoma found the majority of the tracer located in the kidneys as rapid as 2 min p.i and showed little accumulation in the tumour tissue so was not studied further. In 2012, Wuest *et al.* reported the synthesis of [^18^F]-GAZ (63) as a potential PET tracer leverage active transport over the passive diffusion observed with α-5′-[^18^F]-FAZA (57).^169^ Whilst *in vivo* studies in EMT-6 tumour-bearing mice showed uptake in the tumour, the tracer did not accumulate over time resulting in a low, stagnating PET signal over the course of the 60 minute analysis. This was in sharp contrast to α-5′-[^18^F]-FAZA (57), which is retained in hypoxic tissue and continues to accumulate over 60 min p.i. Although uptake was considered poor, the faster clearance rate from the surrounding tissues resulted in a similar SUV (0.66) to α-5′-[^18^F]-FAZA (57) (0.74) 5 minutes p.i. Furthermore, competition studies with 2-[^18^F]-FDG (1) required 1 mM concentrations to observe an effect: this is two orders of magnitude higher than is required with d-glucose (2), suggesting that the uptake mechanism is distinct from that of 2-[^18^F]-FDG (1).

In 2011, the portfolio of tracers was further expanded to include glucosamine derivatives.^170^d-glucosamine has been shown to inhibit tumour growth both *in vitro* and *in vivo*, although the mechanism of inhibition is not fully understood.^171–173^ [^18^F]-FNBG (64) was synthesised and compared *in vivo* in KM tumour-bearing mice with [^18^F]-FAG (24) (Scheme 6). Relative to [^18^F]-FAG (24), [^18^F]-FNBG (64) was found to have a lower tumour to muscle ratio of 5.68 and 4.00 respectively, and poorer accumulation in all organs was noted by comparison. However, the tracer displayed a marginally better tumour to blood ratio (3.79 and 4.40 respectively). Whilst 2-[^18^F]-FDG (1) is generally considered to out-perform glucosamine derivatives with regards to the scope of imaging efficiency, these findings may stimulate interest in delineating the mechanism by which d-glucosamine inhibits tumour growth: this in turn may provide guiding principles to enable the development of more targeted PET agents.

## 8. ^18^F-modified nucleoside tracers

Nucleosides are the constituent building blocks of our genetic blueprint (DNA), and continue to be intensively studied in the context of molecular diagnostic development. They consist of a nucleobase (either a purine or pyrimidine) linked to a pentose subunit by a characteristic β-glycosidic bond. Consequently, this class of small molecules is appealing for the development of novel PET tracers, specifically to monitor tumour-cell proliferation.

### 8.1 Pyrimidine tracers

**Fig. 12:**
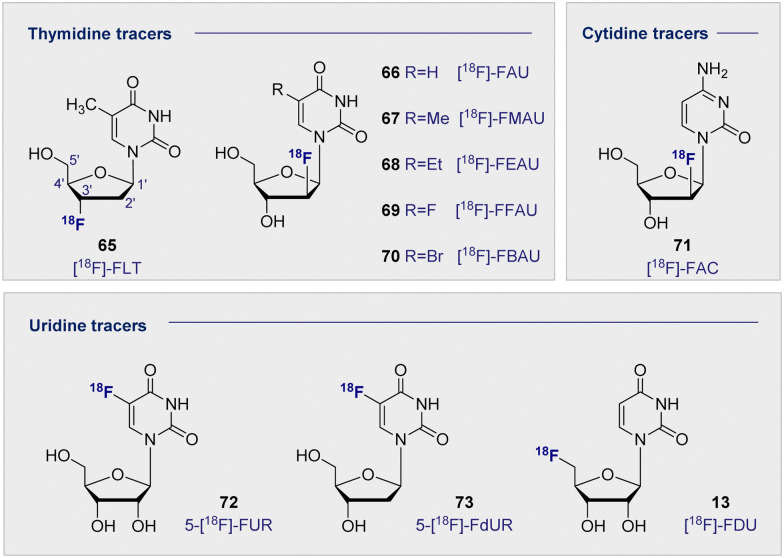
Structures of selected ^18^F-modified pyrimidine-based radiotracers.

Upon inspection of the current suite of ^18^F-nucleoside tracers, it is immediately apparent that the most widely studied system to date is thymidine. This is largely due to it being DNA specific, whilst its de-methylated counterpart uracil is found as its replacement in RNA. In a therapeutic regime, cellular uptake of thymidine tracers is followed by efficient phosphorylation by kinases, such that the now negatively charged species cannot leave the cell. This important feature ensures tracer accumulation and prevents rapid clearance from tumour cells, but does not impact clearance from the blood. It logically follows that the site of ^18^F radiolabelling has been extensively studied on both the sugar and the nucleobase moieties. From the structure-activity data available, ^18^F substitution at the C2′ and C3′ position of the sugar ring is most effective. These regioisomers are characterised by an enhanced stability towards cleavage of the *N*-glycosidic bond by phosphorylases. In the case of the C2 systems, fluorine stereoelectronic effects manifest themselves in a characteristic puckering of the furanose ring.^174–176^ As such the most studied analogues to date have become [^18^F]-FLT (65) and [^18^F]-FMAU (67) (Fig. 12).

[^18^F]-FLT (65) was first developed to address the time constraints imposed with using its predecessor, ^11^C-labelled thymidine, due to its relatively short half-life of 20 min. By comparison, the ^18^F analogue extended the effective life of the compound significantly. This compound undergoes sequential phosphorylations by cytosolic thymidine kinases (TK1 and TK2) to give the triphosphate of FLT. Substitution (C3′OH-^18^F) prevents any further DNA ligation, thus the molecule cannot be further metabolised or incorporated into the DNA, and instead accumulates in proliferating cells. Since increased cell proliferation has been correlated with increased cytosolic thymidine kinase activity, [^18^F]-FLT (65) and other structurally related nucleoside derivatives are promising PET imaging agents.^177^ Furthermore, a close correlation has been observed between antigen Ki-67 (a known nuclear protein associated with cell proliferation) and [^18^F]-FLT (65) uptake in many tumour cell lines, suggesting that an increased accumulation of [^18^F]-FLT (65) could be associated with increased cell proliferation.^178–180^

A clinical trial of 47 patients exhibiting malignant pulmonary nodules was carried out in 2005.^181^ [^18^F]-FLT (65) uptake was exclusive to malignant tumours and enabled successful detection of malignancies in 32 patients. However, false negatives were observed in 6 patients with non-small cell lung carcinoma, pulmonary carcinoid and lung metastases. Further studies carried out between 2003 and 2016 have consistently reported that [^18^F]-FLT (65) has a higher specificity but lower uptake when compared to 2-[^18^F]-FDG (1).^182^ This suggests that whilst less [^18^F]-FLT (65) actually makes it into the cell, the likelihood of the tumour being malignant is higher if uptake occurs. [^18^F]-FLT (65) therefore has an important role to play in the early diagnosis of malignant lesions, particularly if further structural modifications lead to improved cellular uptake.

Related pyrimidine nucleosides in which the 3′-OH is free for DNA ligation and subsequent incorporation have also been investigated. Early studies of [^18^F]-FAU (66) in dogs showed even retention in all organs except for the expected accumulation in the excretion pathway.^183^ Whilst significant accumulation in the bone marrow was expected, due to high concentrations of proliferating tissues, this is where the lowest retention of all was noted. Consequently, further development of [^18^F]-FAU (66) lost momentum and second-generation analogues were introduced (R group substitution, Fig. 12). [^18^F]-FMAU (67), [^18^F]-FEAU (68), [^18^F]-FFAU (69) and [^18^F]-FBAU (70) have been investigated as potential imaging agents for suicide gene expression, in particular herpes simplex virus type 1 thymidine kinase (HSV1-tk) expression.^183–187^ HSV1-tk is a gene that, when expressed, can be the focus of chemotherapy strategies. Suicide genes encode enzymes that can convert pro-drugs into active drugs as part of a therapeutic regime. HSV1-tk, when coupled with ganciclovir, has been successfully used to treat prostate cancer.

Across these studies, tumour uptake was found to be highest when [^18^F]-FFAU (69) was administered and lowest with [^18^F]-FEAU (68) giving rise to the following trend: [^18^F]-FFAU (69) > [^18^F]-FMAU (67) > [^18^F]-FBAU (70) > [^18^F]-FEAU (68). Even though [^18^F]-FEAU (68) led to the lowest uptake, it was found to be preferentially phosphorylated by HSV1-tk and thus visible in colon cancer cells over the wild-type healthy cells. [^18^F]-FMAU (67) is currently in phase I clinical trials, however, poor permeability across the blood–brain-barrier remains a limitation that excludes its use in monitoring cell proliferation in the brain.

A plenum of pyrimidine analogues based on uracil (14) and cytosine have also been investigated. 5-fluorouracil (5-FU) is a known cytostatic agent; however, its toxicity precludes clinical application. It was thought that uridine derivatives might circumvent this toxicity by acting as a pro-drug, being converted into 5-FU once it reached its target. Furthermore, uridine-based analogues confer a range of advantages due to their specificity for RNA over DNA. Like their thymidine DNA counterparts, ^18^F substitution has been investigated on both the sugar and the nucleobase regions. 5-[^18^F]-FUR (72) and its deoxy derivative 5-[^18^F]-FdUR (73) were examined in the 1980s, and a study carried out in AH109A-bearing rats, disclosed that both showed good tumour to blood ratios (3.49 and 6.12 respectively) after 60 min. These high ratios were aided by rapid clearance from the blood and moderate tumour to tissue ratios.^188^

More recently, PET tracers derived from cytosine have become a subject of increasing interest, in particular 1-(2′-deoxy-2′-[^18^F]-fluoroarabinofuranosyl) ([^18^F]-FAC) (71). [^18^F]-FAC (71) was identified from a dCK activity *in vitro* screen of nucleosides to be retained in proliferating cells.^189^ Biodistribution in mice showed accumulation in the thymus and the spleen, to a larger extent than was observed with 2-[^18^F]-FDG (1). Anti-tumour response model studies showed high accumulation in the primary and secondary lymphoid organs, but very little signal in the tumour itself. It has also been determined that [^18^F]-FAC (71) accumulated in autoimmune models but not in viral hepatitis.^190^ This contrasts with 2-[^18^F]-FDG (1), which did not enable discrimination and had similar accumulation in both. A further study investigated the potential of [^18^F]-FAC (71) to visualise brain-infiltrating leukocytes in multiple sclerosis.^191^ Unfortunately, comparable selectivity was not observed in this model as for the hepatitic immunity mouse. This is postulated to be due to brain infiltrating leukocytes dividing slower, which reduces the consumption of high levels of radiotracer. It is pertinent to note that when the chlorinated analogue, 2-chloro-2′-deoxy-2′-[^18^F]-fluoro-9-β-d-arabinofuranosyl-adenine (76) [^18^F]-CFA), was tested in humans, it did not cross the blood–brain barrier.^191^

## 9. ^18^F-modified purine tracers

**Fig. 13:**
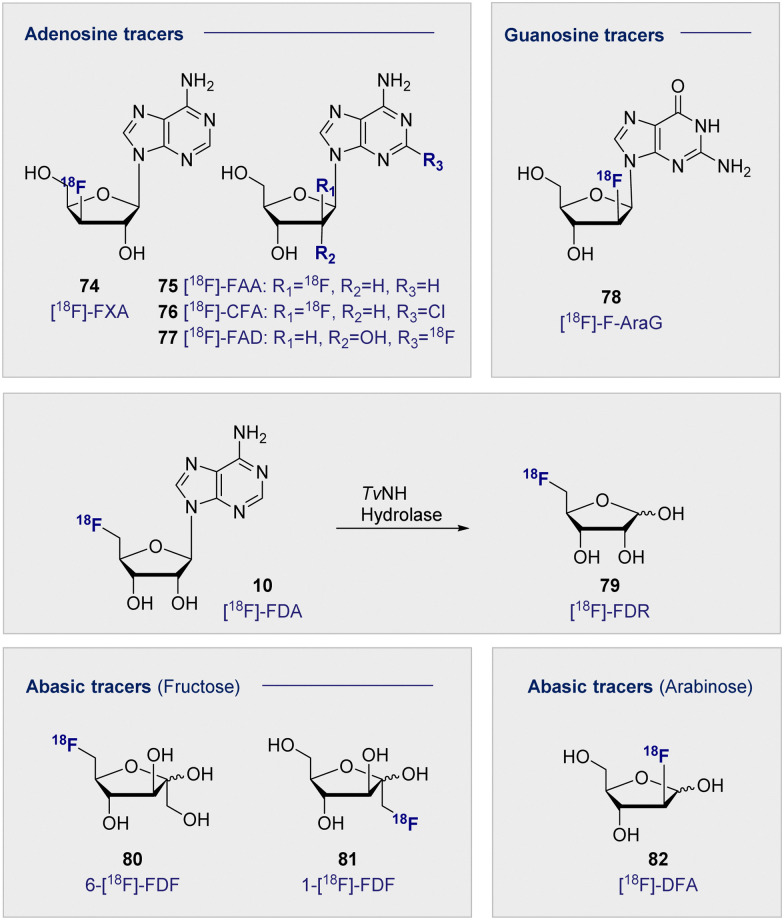
Structure of selected adenosine and guanosine tracers (top). Hydrolysis of 5′-[^18^F]-FDA (10) to [^18^F]-FDR (79) (middle). Structure of selected abasic ribose tracers (bottom).

In analogy to the pyrimidine tracers, substitution at the C3′ on the furanose core was envisaged to promote cellular accumulation by blocking further metabolism through phosphorylation. Fluorinated adenosine analogues have previously been evaluated for anti-viral and anti-tumour activity: C2′ fluorine substituted analogues showed potential in chemotherapeutic development, whereas C3′ substitution facilitated antiviral activity.^192–194^ A comparative study of the two adenosine tracers [^18^F]-FXA (74) and [^18^F]-FAA (75) (Fig. 13, top left), differing only by the site of ^18^F substitution at either the C3′ or C2′ of the sugar, in tumour-bearing mice (human colon cancer xenografts) showed rapid clearance from the blood within the first 20 min.^195^

Accumulation in the heart was postulated to be a result of interaction with adenosine receptors found within the organ, such that the tracer was interacting similar to the native adenosine. Micro-PET studies determined that [^18^F]-FAA (75) accumulated in the tumour tissue, whereas [^18^F]-FXA (74) accumulated predominantly in the heart with no significant signal in the tumour. This strikingly different behaviour is grounded in a single site shift of the ^18^F substituent and further underscores the importance of the structure–function interplay in tracer development.

2-Chloro-2′-deoxy-2′-[^18^F]fluoro-9-β-d-arabinofuranosyladenine (76) ([^18^F]-CFA) has also been recently explored as a tracer for deoxycytidine kinase (dCK) activity.^196^ [^18^F]-CFA (76) showed accumulation in leukemia cells in correlation with dCK expression. This response was also observed in human studies where accumulation was observed in tissues showing high dCK expression *i.e.* secondary lymphoid organs and the hematopoietic bone marrow. This was later extended to image dCK-dependent proliferation in hepatocellular carcinoma (HCC).^197^ Initial biodistribution studies highlighted most uptake in the bladder, liver, kidney and spleen.^198^ As a result, imaging HCC required modifications to combat background signal from healthy liver tissue. This was later achieved through the co-administration of cold ^19^F-CFA.^197^ It is interesting to note the structural similarities of the sugar core between [^18^F]-CFA (76) and the cytosine derivative [^18^F]-FAC (71), given the successful targeting of the same pathway. Substitution on the adenine base has also been explored as a PET tracer for imaging malignancies. 2-[^18^F]-Fluoroadenosine (77) (2-[^18^F]-FAD) was synthesised through a 2-nitroadenosine precursor and injected into Sprague–Dawley rats to obtain biodistribution data.^199^ High accumulation was noted in the lung, heart and kidneys 60 min p.i. Accumulation in the lung was more than 2-fold observed in the heart and kidneys suggesting there could be a specific uptake or receptor that could be exploited for further development in the lungs.

2′-Deoxy-2′-[^18^F]-fluoro-9-β-d-arabinofuranosylguanine (78) ([^18^F]-F-AraG) was advanced for imaging T-cell activation when cell studies confirmed uptake of the tracer in primary T cells from mouse tissue.^200^ This tracer has been effectively deployed to image immune cells in the CNS, where tracer uptake has been shown to be strongly associated with areas of high T-cell concentration in the brain.^201^ As described in Schemes 3, 5′-[^18^F]-FDA (10) can be efficiently prepared through enzymatic synthesis.^50^ This technology has been utilised to prepare the abasic tracer 5-[^18^F]-Fluoro-5-deoxyribose (79) ([^18^F]-FDR) to image tumour-bearing mice (injection of A431 cells).^202^ Specifically, nucleoside hydrolase was harnessed to hydrolyse the base, thereby generating abasic [^18^F]-FDR (79) for use *in vivo* (Fig. 13, centre). In a comparison with 2-[^18^F]-FDG (1), [^18^F]-FDR (79) had a reduced residence time in the tumour, most likely due to lack of trapping from phosphorylation observed with 2-[^18^F]-FDG (1). Importantly, [^18^F]-FDR (79) showed comparable tumour to background contrast (10 min p.i.), rendering this tracer of great interest for further study in tumour lines that cannot be effectively imaged using 2-[^18^F]-FDG (1).

In addition to [^18^F]-FDR (79), other abasic, pentose-based tracers have shown varying degrees of success. Fluorinated fructose analogues 6-deoxy-6-[^18^]fluoro-d-fructose (80) 6-[^18^F]-FDF and 1-[^18^F]Fluorodeoxyfructose (81) (1-[^18^F]-FDF) (Fig. 13, bottom) have been developed to study fructose uptake *via* GLUT2 and GLUT5. Fructose is phosphorylated once in the cell by two distinct mechanisms. This may occur at the C1 position by ketohexokinase (fructokinase) or at the C6 position by hexokinase II. These two processes are noteworthy and provide a degree of orthogonality: blocking the correct site by design enabled either pathway to be elucidated.

GLUT5 has been shown to be overexpressed in certain tumour types, including breast cancer. This is of particular interest as GLUT1 (d-glucose uptake) has been shown to be under-expressed in certain cancers, leading 2-[^18^F]-FDG (1) to be less effective. In this regard, fructose offers potential in tumour tracking as it constitutes an alternative energy pathway to glycolysis.

Radiolabelling fructose at the C1 position to generate 1-[^18^F]-FDF (81) has revealed that uptake can be observed in the kidneys and liver, however rapid clearance and a lack of retention was observed.^203^ 6-[^18^F]-FDF (80) was subsequently synthesised to study imaging in breast cancer in 2009.^204^ This study noted that administration of the tracer led to inhibition of fructose and d-glucose transport, suggesting interaction with GLUT2 and potentially GLUT5. 6-[^18^F]-FDF (80) has been studied in EMT-6 and MCF-7 tumour-bearing mice (breast cancer). The analysis concluded that uptake was not impeded by the co-administration of d-glucose but that it was significantly reduced with co-administration of fructose, suggesting an uptake pathway similar to the native fructose.^205^ Furthermore, when 6-[^18^F]-FDF (80) was incubated with fructokinase, the phosphorylated product was generated, confirming that it is a competent substrate for fructokinase. By contrast, no phosphorylation was observed when incubated with hexokinase II, which can be rationalised on a structural level with the ^18^F label inhibiting enzyme activity. 6-[^18^F]-FDF (80) tumour uptake was observed in EMT-6 tumour-bearing BALB/c mice and was visible 2 h p.i. The experiment was repeated with 2-[^18^F]-FDG (1), which gave similar results although the signal increased over time: the opposite was noted with 6-[^18^F]-FDF (80). At the 2 h time point, accumulation in bone was visible, potentially suggesting defluorination of the tracer. In MCF-7 tumour-bearing mice, the tumour was less visible p.i. with 6-[^18^F]-FDF (80), but the signal did not decrease as rapidly over the 2 h. Although 2-[^18^F]-FDG (1) showed higher retention, 6-[^18^F]-FDF (80) displayed good tumour-selectivity which facilitated image analysis. As recently as 2022, 6-[^18^F]-FDF (80) was evaluated as an agent to image microglia as part of an effort to elucidate the relationship between neuroinflammation and neurodegenerative disease. In a study in LPS injected rodents, increased accumulation was seen in ipsilateral striatum over contralateral (0.985 ± 0.047 and 0.819 ± 0.033 SUV respectively). This result highlighted the potential of 6-[^18^F]-FDF (79) to image the brain's microglial GLUT5 density for further investigation into neurodegenerative disease.^206^

Finally, [^18^F]-2-deoxy-2-fluoroarabinose (82) ([^18^F]-DFA), an arabinose analogue, was evaluated as a ribose salvage probe.^207^ [^18^F]-DFA (82) was successfully deployed to determine the accumulation of ribose in the liver. When administered to mice with metabolic syndrome, a decrease in uptake of [^18^F]-DFA (82) was observed, potentially indicating a correlation between ribose accumulation and d-glucose and/or fat metabolism.

## 10. Conclusions

This *Tutorial Review* is intended to serve as a structure–function guide of glycan-based radiotracers for ^18^F-PET from a chemical perspective. It is envisaged that this reference source will lower the barrier to entry for non-specialists who wish to contribute to this rapidly expanding field of molecular imaging. To further assist in navigating the landscape of this exciting field, a summary appendix of this review is provided (Appendix I). Conventional approaches to radiotracer development are now being complemented by enabling methodologies that range from small molecule strategies through to biocatalysis paradigms: bridging the gap from the laboratory to the clinic remains a decisive factor in achieving success. Further innovation will likely stem from the global production and widespread availability of 2-[^18^F]-FDG (1), which renders it attractive as a carbohydrate building block for practitioners who do not have access to cyclotron facilities.^208^

## Abbreviations

[^18^F]-CFA: 2-Chloro-2′-deoxy-2′-[^18^F]-fluoro-9-β-d-arabinofuranosyl-adenine
[^18^F]-DFA: [^18^F]-2-Deoxy-2-fluoroarabinose
[^18^F]-FDTre: 2-Deoxy-2-[^18^F]-fluoro-d-trehalose
[^18^F]-FAA: 2′-Deoxy-2′-[^18^F]-fluoro-1-β-d-arabinofuranosyl-adenine
[^18^F]-FAraG: 2′-Deoxy-2′-[^18^F]-fluoro-9-β-d-arabinofuranosylguanine
[^18^F]-FAC: 1-(2′-Deoxy-2′-[^18^F]-fluoroarabinofuranosyl)
[^18^F]-FAG: *N*-[^18^F]Fluoroacetylglucosamine
[^18^F]-FAU: 1-(2′-Deoxy-2′-fluoro-β-d-arabinofuranosyl)uracil
[^18^F]-FBAU: 1-(2-Deoxy-2-[^18^F]-fluoro-β-d-arabinofuranosyl)-5-bromouracil
[^18^F]-FDG-2NIm: 1-(2-Deoxy-2-[^18^F]-fluoro-β-d-glucopyranosyl)-2-nitroimidazole
[^18^F]-FDR: 5-[^18^F]-Fluoro-5-deoxyribose
[^18^F]-FEAU: 2-Deoxy-2-[^18^F]-fluoro-1,3,5-tri-O-benzoyl-α-d-ribofuranose
[^18^F]-FFAU: 2′-Deoxy-2′-[^18^F]-fluoro-5-fluoro-1-β-d-arabinofuranosyluracil
[^18^F]-FMAU: 2′-Deoxy-2′-[^18^F]-fluoro-5-methyl-1-β-d-arabinofuranosyluracil
[^18^F]-FNBG: *N*-(2-[^18^F]-Fluoro-4-nitrobenzoyl)glucosamine
[^18^F]-FLT: 3′-Deoxy-3′-[^18^F]fluorothymidine
[^18^F]-FXA: 3′-Deoxy-3′-[^18^F]-fluoro-1-β-d-xylofuranosyl-adenine
[^18^F]-GAZ: *N*-(2-[^18^F]Fluoro-3-(6-*O*-glucosyl)propyl-azomycin
1-[^18^F]-FDF: 1-[^18^F]Fluorodeoxyfructose
1′-[^18^F]-FS: 1′-Deoxy-1′-[^18^F]-fluorosucrose
2-[^18^F]-AFDG: 1,3,4,6-Tetra-acetyl-2-[^18^F]-2-deoxy-fluoro-d-glucose
2-[^18^F]-DFDG: 2-Deoxy-2,2-[^18^F]-difluoro-d-glucose
2-[^18^F]-FDG or [^18^F]FDG: 2-Deoxy-2-[^18^F]-fluoro-d-glucose
2-[^18^F]-FDGal: 2-Deoxy-2-[^18^F]-fluoro-d-galactose
2-[^18^F]-FDM: 2-Deoxy-2-[^18^F]-fluoro-d-mannose
2-[^18^F]-FDT: 2-Deoxy-2-[^18^F]-fluoro-d-talose
3-[^18^F]-FDG: 3-Deoxy-[^18^F]-fluoro-d-glucose
5′-[^18^F]-FDA: 5′-Deoxy-5′ [^18^F]-fluoroadenosine
5-[^18^F]-FdUR: [^18^F]-5-Fluorodeoxyuridine
5-FU: 5-Fluorouracil
5-[^18^F]-FUR: [^18^F]-5-Fluorouridine
6-[^18^F]-FDF: 6-Deoxy-6-[^18^]fluoro-d-fructose
6-[^18^F]-FDG: 6-Deoxy-6-[^18^F]-fluoro-d-glucose
6-[^18^F]-FS: 6-Deoxy-6[^18^F]-fluorosucrose
6′-[^18^F]-FS: 6′-Deoxy-6′ [^18^F]-fluorosucrose
6-[^18^F]-FFuc: 6-[^18^F]-Fluoro-l-fucose
α-5′ -[^18^F]-FAZA: 1-α-d-(5-Fluoro-[^18^F]-5-deoxyarabinofuranosyl)-2-nitroimidazole
αMe-4-[^18^F]-FDG (Me-4FDG): α-Methyl-4-[^18^F]-fluoro-4-deoxy-d-glucopyranoside
β-2′-[^18^F]-FAZA: 1-β-d-(2-Deoxy-2-[^18^F]-fluoroarabinofuranosyl)-2-nitroimidazole
β-3′-[^18^F]-FAZL: 1-β-d-(3-Deoxy-3-[^18^F]-fluorolyxofuranosyl)-2-nitroimidazole
β-5′-[^18^F]-FAZR: 1-β-d-(5-Deoxy-5-[^18^F]-fluororibofuranosyl)-2-nitroimidazole
β-6′-[^18^F]-FAZAL: 1-(6′-Deoxy-6′-[^18^F]fluoro-β-d-allofuranosyl)-2-nitroimidazole
AH109A: Hepatoma cell line
C3H: Mouse strain (high spontaneous frequency of mammary tumours)
ceCT: Contrast-enhanced computer tomography
CNS: Central nervous system
dCK: Deoxycytidine kinase
DNA: Deoxyribonucleic acid
EMT-6: Ephithelial mesenchymal transition-6 (mouse mammary carcinoma cell line)
Et-2′-[^18^F]-FDL: Ethyl-2′-deoxy-2′-[^18^F]-fluorolactose
FITC: Fluorescein isothiocyanate
FMISO: Fluoromisonidazole
Gal-1-P: d-Galactose-1-phosphate
GDP: Guanosine diphosphate
Glucose-6P: Phosphorylated d-glucose
GLUT: d-Glucose transporter
GM1: Monosialotetrahexosylganglioside 1
GM3: Monosialotetrahexosylganglioside 3
GMP: Good manufacturing practice
HIP/PAP: Hepatocarcinoma-intestine-pancreas/pancreatitis-associated protein
HK-II: Hexokinase II
HSV1-tk: Herpes simplex virus type 1 thymidine kinase
L-AAO: l-Amino acid oxidase
lacZ gene: Lactose operon (functional unit of DNA responsible for transport and metabolism of lactose in *E. coli*)
M2: M2 macrophage subtype
MR: d-Mannose receptor (macrophage)
MCF-7: Michigan cancer foundation -7 (breast cancer cell line)
NMR: Nuclear magnetic resonance
PET: Positron emission tomography
p.i.: Post injection
PNP: Purine nucleoside phosphorylase
RCY: Radiochemical yield
RNA: Ribonucleic acid
SAM: *S*-Adenosyl-methyltransferase
SBRT: Stereotactic body radiation therapy
SGLT: Sodium-dependent d-glucose cotransporters
SUV: Standardised uptake value
*t*_1/2_
: Half life
TK1/TK2: Cytosolic thymine kinases
TP: Thymidine phosphorylase
UDP: Uridine diphosphate

## Author contributions

The article was written through contributions from all authors.

## Conflicts of interest

There are no conflicts to declare.

## Supplementary Material

## Appendices

### Appendix I

**Table:**

Compound no.	Name	Target	Labelling Method	Model	Measurement	Comments	Ref.
1	2-[^18^F]-FDG	*d-Glucose metabolism*	Nucleophilic	Rat (Sprague Dawley)	Tumour: muscle ratio (90 min) = 4.75	Gold standard PET tracer, widely effective but difficult to target specific targets due to ubiquity of d-glucose uptake.	68
20	3-[^18^F]-FDG	*d-Glucose metabolism: Brain and heart*	Nucleophilic	Rat (Sprague Dawley)	Tumour: muscle ratio (120 min) = 3.12	Lower uptake than 2-[^18^F]-FDG in heart, liver and kidneys and slower clearance.	68
21	2-[^18^F]-DFDG	*d-Glucose metabolism: Hexokinase activity*	Electrophilic	Mouse (UBC, CD-1)	Brain: blood (60 min) = 2.75	2-[^18^F]-DFDG showed similar ratios to 2-[^18^F]-FDG (Heart: Lung = 12.0) but background uptake in Rhesus monkey much higher for 2-[^18^F]-DFDG.	69
					Heart: lung (120 min) = 9.43		
22	2-[^18^F]-AFDG	*d-Glucose metabolism: GLUT vs diffusion and relationship to hexokinase*	Nucleophilic	Human colon adenocarcinoma cell LS180	—	Increased lipophilicity increased tracer uptake through diffusion in comparison to 2-[^18^F]-FDG. GLUT independent.	70
23	6-[^18^F]-FDG	*d-Glucose transport*	Nucleophilic	Rat (Sprague Dawley)	—	Increased radioactivity observed in skeletal muscle in presence of insulin.	73
24	[^18^F]-FAG	*Tumour detection*	Nucleophilic	KM tumour bearing mice	Tumour: muscle ratio (60 min) = 5.68	Study compared to [^18^F]-FNBG	170
					Tumour: blood ratio (60 min) = 3.79		
25	4-[^18^F]-FDG	*SGLT vs GLUT uptake studies*	Nucleophilic	Mouse Wild type (wt)	Kidney uptake (60 min): 4% ID per g (wt) ∼20% ID per g (*Glut2*-)	Uptake by both SGLT and GLUTs. Accumulates in the brain comparable to 2-[^18^F]-FDG in wt. Accumulated in kidneys in Glut2 knockout.	90
				Glut2 knockout (*Glut2*-)	4% ID per g (*Glut2*-)		
26	α-Me-4-[^18^F]-FDG	*Sodium d-glucose cotransporter (SGLT) activity (high grade astrocytomas)*	Nucleophilic	Human	SUVR_peak_ (tumour) = 1.67	SGLT specific uptake. Low background signal in normal brain tissue allows better imaging.	93
					Signal: noise ratio = 11.5		
6	2-[^18^F]-FDM	*Atherosclerotic plaques*	Nucleophilic	ApoE KO-cuff HFD mice	SUV comparable with 2-[^18^F]-FDG, and sevenfold larger than [^11^C]AM7	2-[^18^F]-FDM determined not to be superior to 2-[^18^F]-FDG in atherosclerotic imaging and binding to MMR was insignificant.	97
		*Hepatocullular carcinoma*	Nucleophilic	AH109A bearing Donryu rats	Tumour: muscle ratio = 5.30	Ratio comparable to 2-[^18^F]-FDG (6.20). However lower uptake in the brain suggests potential as brain tumour imager.	95
27	2-[^18^F]-FDGal	*Hepatoma*	Electrophilic	Hepatoma-bearing C3H mice	Tumour: blood ratio (60 min) = 12.7	Efficient on well differentiated hepatomas but less effective on poorly differentiated hepatomas.	99
					Tumour: muscle ratio (60 min) = 24.2		
28	6-[^18^F]-FDGal	*d-Galactose metabolism*	Nucleophilic	ddY mice	Tumour: muscle ratio (30 min) = 1.73	Mean of three average ratios: (range was 1.02 – 2.55)	106
29	[^18^F]-FDT	*Galactokinase activity and cancer*	Nucleophilic	Fibrosarcoma bearing mice	Tumour: muscle ratio (120 min) = 5.43	Co-administration of tracer with d-galactose inhibited liver uptake of [^18^F]-FDT in rats. Substrate for Galactokinase but not Galacto-1P-uridyltransferase.	109
30	6-[^18^F]-FFuc	*Glycoconjugate synthesis in tumours*	Nucleophilic	Tumour bearing rats: (AH109A, YS, KEG1).	Tumour: brain ratio (120 min) = 9.17	Values of tumour: tissue are an average over five cell lines.	112
				Tumour bearing mice: (FM3A, 3LL).	Tumour: muscle ratio (120 min) = 4.60		112
31	2-[^18^F]-FdFuc	*Energy metabolism*	Nucleophilic	Tumour bearing rats: (AH109A, YS, KEG1).	Tumour: brain ratio = 2.88	Values of tumour: tissue are an average over five cell lines	113
				Tumour bearing mice: (FM3A, 3LL).	Tumour: muscle ratio = 1.33		
32/33	3-[^18^F]Neu5Ac	*Glycoconjugate metabolism in tumours*	Electrophilic	FM3A bearing mice	Tumour: brain ratio (60 min) = 3.71	Tumour uptake ratio was only above 1.0 for brain and muscle. Tracer was quickly excreted to the kidney.	114
					Tumour: muscle ratio (60 min) = 3.71		
34	2,3-di[^18^F]Neu5Ac - C2(ax.)/C3(eq.)	*Glycoconjugate metabolism in tumours*	Electrophilic	FM3A bearing mice	Tumour: brain ratio (60 min) = 10.16	Tumour uptake ratio was above 1.0 for blood, heart, spleen and muscle, Tracer was quickly excreted to the kidney.	114
					Tumour: blood ratio (60 min) = 1.12		
					Tumour: spleen ratio (60 min) = 1.55		
					Tumour: muscle ratio (60 min) = 1.46		
36	[^18^F]-FDS	*Aspergillus fumigatus*	Reduction of 2-[^18^F]-FDG	Immunosuppressed Balb/c mice	Infected muscle: normal muscle (120 min) = 8.90	Values as high as 30.7 fold higher uptake observed in infected over normal tissue.	80
37	2′-[^18^F]-FDL	*β-Galactosidase activity*	Enzymatic	Rosa-26 mice	—	Does not cross the cell membrane and rapidly excreted through the bladder.	116
38	Et-2′-[^18^F]-FDL	*Pancreatic carcinoma*	Nucleophilic	*Ex vivo* mouse tissue (pancreatic carcinoma)	—	Tracer binds in regions highly correlated with HIP/PAP expression and inhibited by lactose administration.	117
39	1′-[^18^F]-FEL	*Pancreatic carcinoma*	Nucleophilic	Mouse (Nude, bearing)	Peritumoral tissue: muscle ratio = 12.64	Selective for peritumoral tissue over background pancreatic tissue.	120
40	1′-[^18^F]-FS	*Maize sucrose transporter ZmSUT1*	Nucleophilic	Maize plants (zmsut1-m1 mutant)	—	Successfully translocated	123
41	6′-[^18^F]-FS	*Maize sucrose transporter ZmSUT1*	Nucleophillic	Maize plants (zmsut1-m1 mutant)	—	Successfully translocated	123
42	6-[^18^F]-FS	*Maize sucrose transporter ZmSUT1*	Nucleophilic	Maize plants (zmsut1-m1 mutant)	—	Successfully translocated	123
43	[^18^F]-FDTre	*Mycobacterium tuberculosis*	Chemoenzymatic	*M. smegmatis*	—	Uptake was reported in a linear and time dependent manner. By comparison mammalian cells showed no appreciable uptake.	127
44	6-[^18^F]-Fluoromaltose	*Pathogenic bacteria*	Nucleophilic	Nude mice (infected with *Escherichia coli*)	—	Tracer accumulation was 2-fold higher in leg injected with bacteria than the healthy leg at all three time points measured. Tracer specific for bacterial cells.	133
45	1-[^18^F]-Fluoromaltose	*Pathogenic bacteria*	Nucleophilic	*Escherichia coli* (ATCC33456) (bacterial) and EL4 (mammalian)	—	^14^C labelled maltose uptake was blocked by co-administration of the tracer.	136
48	[^18^F]-Fluoromaltohexaose	*Pathogenic bacteria*	Nucleophilic	*Escherichia coli Escherichia coli (LamB mutant), mammalian cells (hepatocytes)*	—	More efficient at bacterial imaging than 2-[^18^F]-FDG. Selective uptake in *E. coli* over mutant strain and mammalian cells.	139
49	6′′-[^18^F]-Fluoromaltriose	*Pathogenic bacteria*	Nucleophilic	Nude mice infected with *Escherichia coli*	—	Tracer was accumulated in infected muscle 3.5 fold in comparison to un-infected contralateral muscle	140
50	[^18^F]AAX21	*Pathogenic bacteria*	Nucleophilic followed by click chemistry	Blood serum (human)	—	Stable in human blood serum (>120 min)	142
51	[^18^F]-GM1	*Neurodegenerative disease*	Nucleophilic	Cynomolgus monkey (Non-human primate brain)	SUV (brain) > 0.4 Brain: blood ratio = 0.03	Low uptake in the brain compared with previous studies. High uptake in the heart could lead to complications with vascular insulin resistance and heart failure.	146
57	α-5′-[^18^F]-FAZA	*Hypoxia*	Nucleophilic	Human (high-grade glioblastoma)	Tumour: brain ratio = 5.3	Average over 7 patients. High ratio aided by lack of tracer accumulation in normal brain tissue.	164
58	β-5′-[^18^F]-FAZR	*Hypoxia*	Nucleophilic	HCT 116 colorectal carcinoma cells	—	Poor interaction with five hNTs suggests β-5′-[^18^F]-FAZR is not transported into human cells efficiently.	165
59	β-6′-[^18^F]-FAZAL	*Hypoxia*	Nucleophilic	EMT6 tumour bearing NMRI-*Foxn1* mice	Tumour: muscle ratio (120 min) = 2.13	Ratio from hypoxic conditions in comparison to normoxic at 1.22.	166
60	β-2′-[^18^F]-FAZA	*Hypoxia*	Nucleophilic	—	—	—	167
61	β-3′-[^18^F]-FAZL	*Hypoxia*	Nucleophilic	—	—	—	167
62	[^18^F]-FDG-2-NIm	*Hypoxia*	Nucleophilic	Wistar rats injected with Walker 256 rat mammary carcinoma cells	Uptake in tumour = 0.43 ± 0.09% ID per g	Does not accumulate in tumours or hypoxic tissue	168
63	[^18^F]-GAZ	*Hypoxia*	Nucleophilic	EMT6 tumour bearing Balb/C mice	Tumour: muscle ratio (60 min) = 1.87	Evidence suggests not transported in the presence of d-glucose.	169
64	[^18^F]-FNBG	*Tumour detection*	Nucleophilic	KM tumour bearing mice	Tumour: muscle ratio (60 min) = 4.00 Tumour: blood ratio (60 min) = 4.40	Study compared to [^18^F]-FAG	170
65	[^18^F]-FLT	*Lymphoma*	Nucleophilic	Human	SUV(Aggressive lymphoma) = 5.9	Aggressive lymphoma patients (21)	180
					SUV(Indolent lymphoma) = 2.3	Indolent lymphoma (11)	
66	[^18^F]-FAU	*DNA synthesis*	Nucleophilic	Dog	Tissue: blood ratio = ∼1.0	[^18^F]-FAU evenly distributed to most organs except higher ratio in excretory pathways (kidney and gallbladder)	183
					Tissue: muscle ratio = >1.0		
67	[^18^F]-FMAU	*DNA synthesis*	Nucleophilic	Human patients: prostate cancer	Tumour: background ratio = 6.31	High tumour: background ratios a combination of efficient uptake in the tumours and poor uptake in surrounding tissue *e.g.* brain, bone marrow and pelvis.	186
				Breast Cancer	Tumour: background = 5.43		
				Recurrent Glioma	Tumour: background = 2.91		
68	[^18^F]-FEAU	*DNA synthesis*	Nucleophilic	HSV-tk tumour bearing nude mice	—	Low uptake, however preferential phosphorylation improves visibility in colon cancer cells.	187
69	[^18^F]-FFAU	*DNA synthesis*	Nucleophilic	HSV-tk tumour bearing nude mice	—	Accumulates significantly in HSV-tk tumours only.	184
70	[^18^F]-FBAU	*DNA synthesis*	Nucleophilic	HSV-tk tumour bearing nude mice	Tumour: blood ratio (120 min) = 9.6	Lower uptake than [^18^F]-FMAU	209
71	[^18^F]-FAC	*Deoxyribonucleotide salvage pathway*	Nucleophilic	C57/BL6 mice	Uptake in spleen = 2.16 ± 0.48% ID per g	Uptake higher than 2-[^18^F]-FDG in spleen. (Not detected in thymus)	189
					Uptake in thymus = 3.29 ± 0.15% ID per g		
72	5-[^18^F]FUR	*Nucleic acid metabolism*	Electrophilic	AH109A tumour bearing rats	Tumour: blood ratio (60 min) = 3.49	Rapid clearance from the blood facilitated improved imaging.	188
73	[^18^F]FdUR	*Nucleic acid metabolism*	Electrophilic	AH109A tumour bearing rats	Tumour: blood ratio (60 min) = 6.12	Rapid clearance from the blood facilitated improved imaging.	188
74	[^18^F]-FXA	*Cell proliferation and gene expression*	Nucleophilic	HSV-tk tumour bearing nude mice	Tumour: blood ratio (120 min) = 0.47	Not a suitable tracer for tumour imaging, appears not to be a substrate for HSV-tk.	195
					Heart: blood ratio (120 min) = 8.15		
75	[^18^F]-FAA	*Cell proliferation and gene expression*	Nucleophilic	HSV-tk tumour bearing nude mice	Tumour: blood ratio (120 min) = 2.92	Moderate tracer for tumour imaging, appears not to be a substrate for HSV-tk.	195
					Spleen: blood ratio (120 min) 21.89		
76	[^18^F]-CFA	*Deoxyribonucleotide salvage pathway*	Nucleophilic	Human	Absorbed dose co-efficient: Bladder = 2.34E-01 mGy/MBq	Safe administration in humans, no adverse side effects. Uptake most prominent in bladder, liver, kidneys and spleen.	198
					Liver = 2.62E-02 mGy/MBq		
					Kidneys = 2.51E-02 mGy/MBq		
77	[^18^F]-FAD	*A*_*1A*_ *, A*_*2A*_ *, A*_*2B*_ *and A*_*3A*_ *Adenosine receptors.*	Nucleophilic	Rats (Sprague-Dawley)	Uptake in lung = 9.15 ± 0.15% ID per g	Biodistribution studies showed decreasing uptake from lung > kidney > heart > spleen.	199
78	[^18^F]-F-AraG	*Multiple sclerosis lesions (via their T cell accumulation)*	Nucleophilic^200^	C57BL/6J mice	Brain: blood ratio (50 min) = 1.1 ± 0.2	[^18^F]-F-AraG crosses the blood brain barrier.	201
10	5′-[^18^F]-FDA	*—*	Enzymatic	—	—	Used as a precursor to [^18^F]-FDR.	51
79	[^18^F]-FDR	*Tumour imaging*	Hydrolysis of 5′-[^18^F]-FDA^202^	A431 tumour bearing mouse	—	[^18^F]-FDR has more rapid uptake than 2-[^18^F]-FDG 5 min p.i. Stable for 20 min before signal decreases.	202
80	6-[^18^F]-FDF	*Breast cancer*	Nucleophilic	EMT-6 and MCF-7 tumour bearing BALB/c mice	EMT-6 tumour bearing mice: Tumour: muscle ratio (30 min) = 2.51	2-[^18^F]-FDG showed greater accumulation in MCF-7 mice but 6-[^18^F]-FDF good selectivity for the tumour.	205
81	1-[^18^F]-FDF	*Fructose uptake*	Nucleophilic	C3H fibrosarcoma bearing mice	Tumour: blood ratio (120 min) = 1.45	Uptake in the kidneys and liver, but rapid clearance leads to poor accumulation.	203
82	[^18^F]-DFA	*Ribose salvage*	Nucleophilic	C57BL/6 mice	—	Greatest accumulation observed in the kidney, liver and intestines	207
